# Serum NT/CT SIRT1 ratio reflects early osteoarthritis and chondrosenescence

**DOI:** 10.1136/annrheumdis-2020-217072

**Published:** 2020-07-14

**Authors:** George Batshon, Jinan Elayyan, Omar Qiq, Eli Reich, Louisa Ben-Aderet, Leonid Kandel, Amir Haze, Jürgen Steinmeyer, Veronique Lefebvre, Hong Zhang, Jennifer Elisseeff, Yves Henrotin, Ali Mobasheri, Mona Dvir-Ginzberg

**Affiliations:** 1 Institute of Dental Sciences, Hebrew University of Jerusalem, Jerusalem, Israel; 2 Joint Replacement and Reconstructive Surgery Unit, Orthopaedic Surgery Complex, Hadassah Mount Scopus Hospital, Jerusalem, Israel; 3 Laboratory for Experimental Orthopaedics, Dept. of Orthopaedics, Justus Liebig University Giessen, Gießen, Germany; 4 Developmental Biology Research Affinity Group, Children’s Hospital of Philadelphia, Philadelphia, Pennsylvania, USA; 5 Translational Tissue Engineering Center, Wilmer Eye Institute and the Department of Biomedical Engineering, Johns Hopkins University, Baltimore, Maryland, USA; 6 Bone and Cartilage Research Unit, Arthropole Liège, Institute of Pathology, University of Liège, Liege, Belgium; 7 Research Unit of Medical Imaging, Physics and Technology, Faculty of Medicine, University of Oulu, Oulu, Finland

**Keywords:** osteoarthritis, chondrocytes, inflammation, TNF-alpha

## Abstract

**Objective:**

Previous work has established that the deacetylase sirtuin-1 (SIRT1) is cleaved by cathepsin B in chondrocytes subjected to proinflammatory stress, yielding a stable but inactive N-terminal (NT) polypeptide (75SIRT1) and a C-terminal (CT) fragment. The present work examined if chondrocyte-derived NT-SIRT1 is detected in serum and may serve as an investigative and exploratory biomarker of osteoarthritis (OA).

**Methods:**

We developed a novel ELISA assay to measure the ratio of NT to CT of SIRT1 in the serum of human individuals and mice subjected to post-traumatic OA (PTOA) or age-dependent OA (ADOA). We additionally monitored NT/CT SIRT1 in mice subject to ADOA/PTOA followed by senolytic clearance. Human chondrosenescent and non-senescent chondrocytes were exposed to cytokines and analysed for apoptosis and NT/CT SIRT1 ratio in conditioned medium.

**Results:**

Wild-type mice with PTOA or ADOA of moderate severity exhibited increased serum NT/CT SIRT1 ratio. In contrast, this ratio remained low in cartilage-specific *Sirt1* knockout mice despite similar or increased PTOA and ADOA severity. Local clearance of senescent chondrocytes from old mice with post-traumatic injury resulted in a lower NT/CT ratio and reduced OA severity. While primary chondrocytes exhibited NT/CT ratio increased in conditioned media after prolonged cytokine stimulation, this increase was not evident in cytokine-stimulated chondrosenescent cells. Finally, serum NT/CT ratio was elevated in humans with early-stage OA.

**Conclusions:**

Increased levels of serum NT/CT SIRT1 ratio correlated with moderate OA in both mice and humans, stemming at least in part from non-senescent chondrocyte apoptosis, possibly a result of prolonged inflammatory insult.

**Key messages**

**What is already known about this subject?**

- We have previously shown that SIRT1 can be cleaved in cartilage during osteoarthritis (OA) and under inflammatory conditions. It is also known to be detected in sera in various diseases, as we detail in the Discussion section within the manuscript text.

**What does this study add?**

- This study adds a new method for detecting Sirt1 cleaved variants in sera. We show that these variants are directly linked to cartilage degeneration, using our genetic mouse models which bear ablated *Sirt1* from cartilage. These mouse models (ie, with ablated *Sirt1* from cartilage) subjected to ageing or post-traumatic OA show reduced levels of these variants in sera, despite OA development. Another novelty is the association of these cleaved Sirt1 variants with chondrosenesence, which has not been previously reported. In fact, eliminating chondrosenescent cells reduced Sirt1 variants in vitro and in vivo.

**How might this impact on clinical practice or future developments?**

- The data herein merit clinical studies to validate this serum measure for categorising and stratifying OA severity, which can accelerate drug development and personalised medicine for OA sufferers. Importantly, the portion related to chondrosenescence and senolytic drug administration indicate that this serum measure can predict senolytic drug efficiency in OA, which can minimise the need for imaging modalities to assess joint structural changes following drug administration, to ultimately develop disease-modifying OA drugs for clinical use.

## Background

Articular cartilage is a thin hyaline tissue covering opposing bone ends in synovial joints. It prevents bone end erosion by absorbing mechanical loads inflicted by daily activities and ensures friction-less and pain-free joint articulation and movement.^1–3^ Osteoarthritis (OA), the most prevalent degenerative joint disease in humans, is characterised by progressive deterioration and loss of articular cartilage. Clinical symptoms typically arise only once cartilage is already irreversibly destroyed. They include pain, radiographic features and joint effusion of exposed subchondral bone.^1 2^

Cartilage destruction leading to OA is due to excessive mechanical stress to the joint and to proinflammatory insults that suppress the anabolic activities of chondrocytes and evoke catabolic activities.^1^ Chondrosenescence was recently cemented as a hallmark of OA pathogenesis, by showing that the clearing senescent cells from articular cartilage was beneficial to prevent- OA.^4 5^ Chondrosenescence increases with age as well as during post-traumatic joint insults. It contributes to gradual tissue loss as chondrocytes exhibiting a senescence-associated secretory phenotype (SASP), which exude proinflammatory cytokines that may affect non-senescent neighbouring cells.^6 7^

Nowadays, a main challenge in the management of OA is to develop disease-modifying drugs capable of halting disease progression, reversing tissue damage, restoring structure and relieving symptoms. Another major challenge is to identify effective biomarkers that meet the ‘BIPED’ biomarker classification criteria, established by the National Institutes of Health Osteoarthritis Biomarkers Network; including biomarkers for Burden of disease (B), Investigative (I), Prognostic (P), Efficacy of intervention (E) and Diagnostic (D).^8 9^ Notably, while diagnostic markers help determine the disease stage, prognostic markers help predict the rate and severity of disease progression.^10 11^ Such markers would be instrumental to assess optimal timing for candidate drug treatments and to reduce the need for imaging modalities (ie, MRI or X-ray).

Accumulating reports support the notion that SIRT1, a- nicotinamide adenine dinucleotide (NAD)-dependent enzyme silent information regulator 2 type 1 deacetylase, is critical in maintaining adult cartilage health by promoting chondrocyte survival and extracellular matrix (ECM) homeostasis.^12^ Loss of SIRT1 activity has been described in several age-related diseases, including Alzheimer’s^13^ as well as diabetes type 2,^14^ metabolic syndrome and obesity,^15 16^ and SIRT1 circulating levels have recently become attractive biomarkers of such conditions.^17–19^ Mounting data support that SIRT1 is proteolytically inactivated during OA.^20 21^ Thus, in this study, we developed an ELISA assay that distinguishes a SIRT1 N-terminal polypeptide (NT) from SIRT1 C-terminal polypeptides (CT) in human and mouse serum. We used this assay to assess if serum NT/CT SIRT1 ratio is elevated in mice and humans with moderate OA and could therefore be used as a potential disease biomarker at an early, yet asymptomatic stage of joint deterioration.

## Materials and methods

Online supplementary S1 contains all sections materials and methods.

10.1136/annrheumdis-2020-217072.supp1 Supplementary data

## Results

### Development of ELISA to measure the serum NT/CT SIRT1 ratio

Our group initially described a CT truncation in human and mouse SIRT1 carried out by cathepsin B,^20^ which docks to an unstructured loop domain of SIRT1.^22^ This results in a human 75 kDa NT-intact Sirt1 polypeptide with impaired deacetylase activity.^21–24^ Based on these data, it is envisioned that NT SIRT1 antibodies would detect both 75SIRT1 and flSIRT1 given that both possess intact NT domains. In 2012, a report by Chalkiadaki and Guarente showed that HFD resulted in NT-domain cleavage of Sirt1, by caspase 1, resulting in a similar 75 kDa polypeptide with intact CT, in inflamed adipose tissue.^25^ To this end, here, we aimed to assess both NT and CT domains of SIRT1 to differentiate between OA severity and other conditions related to fat metabolism or other unknown conditions causing an NT truncation.

**Figure 1:**
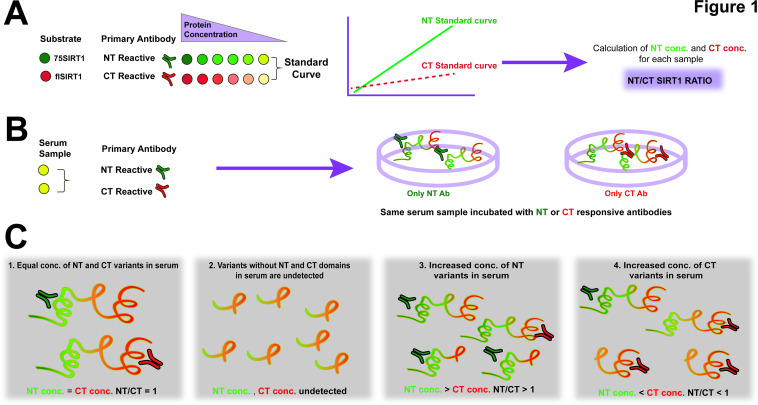
ELISA assay for N-terminal (NT)/C-terminal (CT) SIRT1 ratio. Diluted serum or conditioned media are incubated with either NT-reactive or CT-reactive antibodies and concentrations are calculated based on the respective standard curve. The plate configuration is added next to the standard curve illustration (A). Each serum sample is either incubated with an NT-reactive or CT-reactive antibody (B), wherein ratio between the NT and CT concentrations denoted NT/CT SIRT1 ratio. (C) Possible scenarios related to this ELISA method: (1) an equal NT and CT ratio of 1 indicates that there is no increase in either protein domains of SIRT1 in examined samples; (2) cleavage of both NT and CT domains will render undetected; (3) increased NT concentration as compared with CT concentration rendering a value over 1; (4) increased CT concentration as compared with NT concentration rendering a value less than 1. Notably, using this method, one cannot distinguish between full length and cleaved variants of SIRT1, rather NT-reactive or CT-reactive polypeptides of SIRT1.

Accordingly, we hypothesised that in OA, degenerated cartilage would contribute to increased NT SIRT1 in serum. This assay can either measure intact SIRT1 and/or SIRT1 polypeptides bearing either CT (aa 700-747) or NT (aa 1-131), as illustrated in the plate set-up in figure 1A, B, and in the predicted assay outcomes in figure 1C. Notably, a ratio of 1 would indicate equal concentrations of NT and CT SIRT1 polypeptides, which may not necessarily be the full molecular length of SIRT1. The ELISA assay was validated for reproducibility via %CV assay, specificity via spike in assay and linearity via dilution recovery (table 1) and western blot confirmed the capacity of antibodies to recognise full length or 75SIRT1 (online supplementary S2).

**Table 1:** ELISA validation parameters

Mice sera	NT antibody	CT antibody	Human sera	NT antibody	CT antibody
**%CV**	26.3	18.35	**%CV**	18.5	28.9
**Spike in**	88.6%±9%	96%±3.5%	**Spike in**	102%±12.8%	89.8%±7.0%
**Dilution recovery**	122%±64%	123%±19%	**Dilution recovery**	149%±18%	139%±10%
**Antibody epitope**	1-131aa	728-737aa	**Antibody epitope**	1-131aa	700-747aa
**Antibody manufacturer** (**cat#**)	Millipore (07–131)	Cell signalling (2028)	**Antibody manufacturer**	Millipore, (07–131)	Bethyl laboratories (A300-688)

(1) Percent coefficient of variance (%CV) was calculated for N-terminal (NT) and C-terminal (CT) against mouse and human epitopes, and was calculated for five different serum samples in three identical plates. The mean concentration for a given samples was determined and divided by the SD. Values were then multiplied by 100 to achieve %CV values. (2) Dilution recovery for NT and CT against mouse and human epitopes was calculated for three different serum samples (n=3), each individually diluted 1:1000 (A) or 1:2000 (B), which is within the working dilution range. Dilution recovery was calculated by ((A)/(B*2))*100. (3) Spike in: (a) human samples: For NT/CT antibody, the neat serum samples was diluted 1:5000 and spiked with 3.1 ng/mL. After calculating the protein conc. via standard curve, the neat values were subtracted from the spiked and divided by the expected concentration, which was multiplied by 100 to reach % spiked in recovery; (b) mouse samples spike in for NT antibodies were done in 1:3000 (neat) spiked with 1.5 ng/mL flSirt1 (n=5). Spike in for mouse CT antibodies was done in 1:5000 (neat) spiked with 3.1 ng/mL flSirt1 (n=3). Last two rows in table include epitope recognition and manufacturer information.

### The serum NT/CT SIRT1 ratio is elevated in PTOA-induced and aged mice

**Figure 2:**
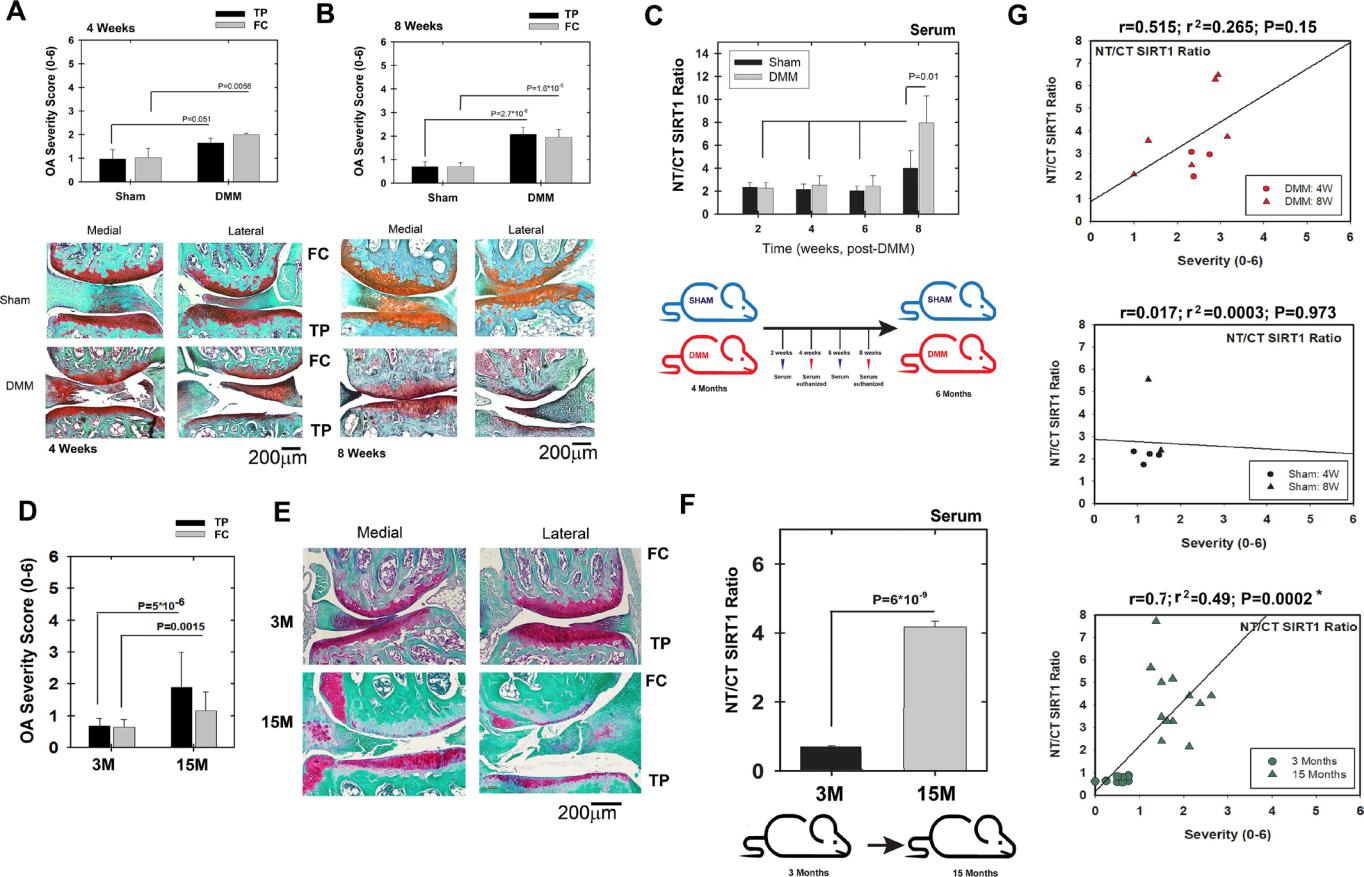
Increased N-terminal (NT)/C-terminal (CT) SIRT1 ratio is observed in moderate osteoarthritis (OA) severity in wild-type (WT) mice. WT (male and female) Sirt1 mice 3M were subjected to destabilisation of the medial meniscus (DMM) or sham procedures (see the Materials and methods section, supplemental text 1) and euthanised at 4 and 8 weeks post DMM. Joints were harvested and processed for histology (Safranin O/Fast green staining) and ranked for OA severity based on Glasson *et al* 30, for tibial plateau (TP) or femoral chondyle (FC). Representative sections for each time point are presented underneath each OA ranking diagram. (A) 4 weeks: sham, n=7; DMM, n=6. (B) 8 weeks: sham, n=8; DMM, n=10. (C) Indirect ELISA of serum derived from WT sham and DMM mice (time points 2, 4, 6 and 8 weeks post procedure, n=7). Below the graph is a scheme of the experimental set-up: (D) WT (female) Sirt1 mice 3M and 15M were euthanised, joints harvested and processed for histology (Safranin O/Fast green staining) and ranked for OA severity based on Glasson *et al.* 30 (E) Representative sections for each time point (n=8). (F) Indirect ELISA of serum derived from 3M and 15M mice (n=6). Below the graph is a scheme of the experimental set-up. Statistical significance is indicated by an asterisk (*, p<0.05) based on Mann-Whitney analysis. (G) Correlations of NT/CT SIRT1 ratio versus OA severity per individual mouse within the DMM (upper graphs); sham (middle graph) and ageing cohorts (lower graph). Correlations are presented as Pearson’s correlation (r; 1 being the best linear fit and 0 being the weakest fit), linear regression (r^2^) and p value.

To assess if SIRT1 fragments are affected by OA, we performed DMM surgery on wild-type (wt) mice (CD1/129/J) to induce post-traumatic osteoarthritis (PTOA, figure 2A, B) and we also compared young (3 months) and old (15 months) mice (figure 2D, E). In mice with PTOA, both the tibial plateau (TP) and the femoral condyles (FC) showed a significant increase in OA severity compared with mice subjected to sham surgery (figure 2A, B, respectively), with severity in the mild range. While no difference in the serum NT/CT SIRT1 ratio was detected at 2, 4 and 6 weeks post surgery, a significant increase of approximately twofold was measured at 8 weeks in mice with DMM compared with controls (figure 2C, below panel illustration of experimental set-up). Assessing the correlation between OA severity and NT/CT SIRT1 ratio of individual mice (figure 2G) shows higher correlation values in DMM (upper panel) versus sham mice (middle panel), indicating that PTOA is associated with increased NT/CT SIRT1 ratio.

We next examined if age-induced OA causes changes in serum NT/CT SIRT1 ratio, by comparing 3M and 15M mice (figure 2D, E). The results show an approximately threefold increase in OA severity in the TP and FC on ageing (figure 2D), accompanied with an approximately fourfold increase in serum NT/CT SIRT1 ratio (figure 2F, below panel illustration of experimental set-up). In conclusion, an increase in the serum NT/CT SIRT1 ratio is thus associated with both early-stage PTOA and aging-related OA. Lower panel of figure 2G shows a significant correlation of OA severity with NT/CT SIRT1 ratios in the ageing mice cohort.

### The serum NT/CT SIRT1 ratio is elevated in human patients with OA

**Figure 3:**
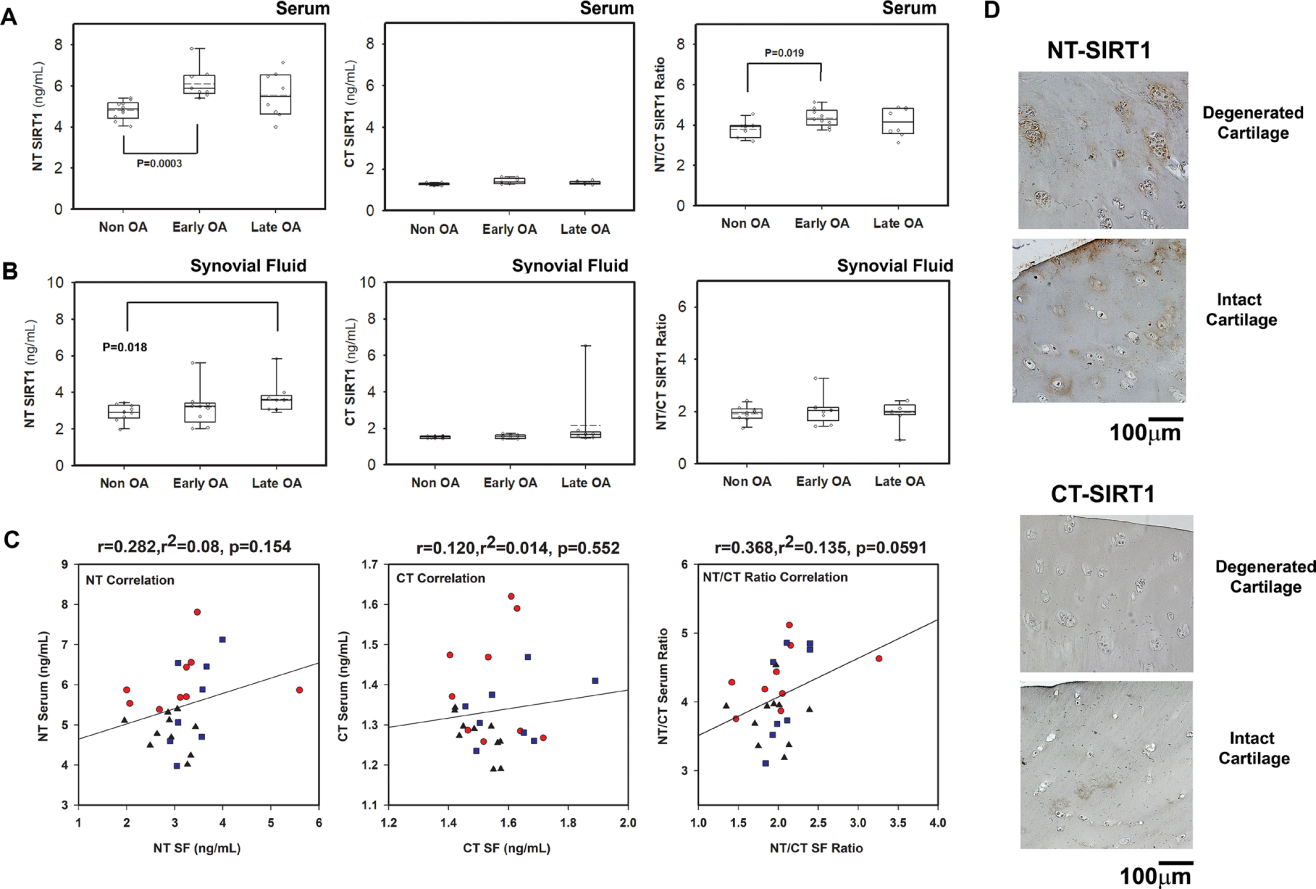
Degenerating cartilage explants and patients with early osteoarthritis (OA) display elevated N-terminal (NT)/C-terminal (CT) SIRT1 ratio. (A) Indirect ELISA of individual human serum samples derived from patients with non-OA, early OA (outerbridge score <2) and late OA (outerbridge score >2), (n>9) for either NT-SIRT1, CT-SIRT1 (ng/mL) or NT/CT SIRT1 ratio. (B) Indirect ELISA of individual human synovial fluid (SF) samples derived from patients with non-OA (n=10), early OA (outerbridge score<2, n=9) and late OA (outerbridge score>2), (n=9) for either NT-SIRT1, CT-SIRT1 (ng/mL) or NT/CT SIRT1 ratio. Median in black bar and average in broken black bar within the box (A–C). Whiskers represent first and third quartile sample values. (C) Serum and SF correlation analysis for NT, CT and NT/CT SIRT1 ratio. Correlations are presented as Pearson’s correlation (r; 1 being the best linear fit and 0 being the weakest fit), linear regression (r^2^) and p value. (D) Immunohistochemistry for NT-SIRT1 and CT-SIRT1 of OA cartilage (n=5). Statistical significance is indicated by an asterisk (*) (p<0.05) based on Mann-Whitney test.

The analysis of a cohort of 28 patients (figure 3A) showed an increase in the serum NT/CT SIRT1 ratio in individuals with early OA compared with healthy donors, mostly due to higher NT values (NT SIRT1: p=0.0003 non-OA vs early OA; NT/CT SIRT1: p=0.019 non-OA vs early OA). Interestingly, NT values are increased in synovial fluid between healthy donors and late OA (SF; p=0.018; figure 3B). Correlation data of each variant in serum and SF shows a stronger correlation of the NT variants in serum and SF, versus CT variants (figure 3C, left and middle panel). Moreover, the strongest correlation (p=0.059) was observed for the NT/CT SIRT1 ratio when assessing serum and SF, using an indirect ELISA method (figure 3C, right panel). These data possibly indicate that NT variants can efficiently reach the circulation during the early stages of OA, while their constant production at late stages of the disease may tend to accumulate in SF. Cumulatively, the results support the notion that serum NT/CT SIRT1 ratio is predictive of early OA and may arise from synovial fluid.

To further assess if either NT, CT or NT/CT are associated with risk factors of OA, we carried out correlation assays for serum, which display similar significant correlations of all three variables to age (online supplementary S3A), and weaker correlations to body mass index (BMI) and OA severity. Based on these data, we could not conclude that one variable is preferable over others for assessing OA severity. However, it appears that among three variables NT presented the strongest correlation to OA severity (online supplementary S3A table), using an indirect ELISA method.

Assessing the same cohort with a sandwich ELISA method (online supplementary S3B, C) displayed significant increase for NT SIRT1 with OA severity, which was marginally significant for CT and NT/CT SIRT1 variants, the latter due to reduced sensitivity of the assay to CT SIRT1 variants. Moreover, while significant correlations for NT SIRT1 were found with age, BMI and OA severity, CT and NT/CT SIRT1 values did not correlate significantly with age, BMI and OA severity. The sandwich ELISA platform appears to favour assessment of NT variants; however, this remains to be fully determined with a more optimised assay that enables better detection of CT SIRT1 variants.

We next assessed degenerative and intact cartilage regions for NT and CT SIRT1 levels. We observed a more enhanced staining for NT-SIRT1 in degenerated zones than for CT-SIRT1 (figure 3D), supporting that NT-SIRT1 fragments are generated by articular chondrocytes as OA progresses (n=5, end-stage OA samples). Accordingly, we previously showed that protein extracts from degenerated cartilage contained higher levels of 75SIRT1 than intact cartilage sites.^24^ Taken together, these data indicated that chondrocytes in human OA cartilage produce elevated levels of NT-intact SIRT1 fragments, which are at least partially released in the circulation and can therefore serve as indicators of OA.

### Chondrocyte-specific inactivation of Sirt1 does not alter skeletal development in mice

Given that SIRT1 is a ubiquitous protein, its fragments detected in serum could be derived from other tissues than cartilage. Indeed, others have shown that the level of 75SIRT1 is detected in tissue undergoing endothelial senescence,^26^ ocular inflammation^27^ and macrophage activation.^28^ To this end, we next asked if the changes observed in the serum NT/CT SIRT1 ratio during OA progression originated from chondrocytes.

As a first step, we generated doxycycline-inducible chondrocyte-specific *Sirt1* knockout mice (*ATC^Cre^Sirt1^fl/fl^*,^29^), which we compared with *Sirt1^fl/fl^* controls. To assess if there is a growth phenotype in mutant mice, we induced the embryos by doxycycline and confirmed that skeletal growth was unaffected by *Sirt1* genetic ablation (online supplementary S4), as previously reported in Col2a1 conditional mice of Sirt1.^12^ Costal chondrocytes were isolated from embryos and confirmed for lack of Sirt1 protein and reduced activity as compared with *Sirt1^fl/fl^* littermates (online supplementary S5; panels A and B). Furthermore, *ATC^Cre^Sirt1^fl/fl^* and *Sirt1^fl/fl^* were grown to reach 3 months and induced with doxycycline for 2 weeks. SIRT1 protein levels were detected in brain, muscle, fat and heart tissues among the genotypes, as observed in immunoblots (online supplementary S5C), supporting that recombination did not occur in these tissues and likely specific to cartilage, as previously reported.^29^

### Serum SIRT1 variants are cartilage derived

**Figure 4:**
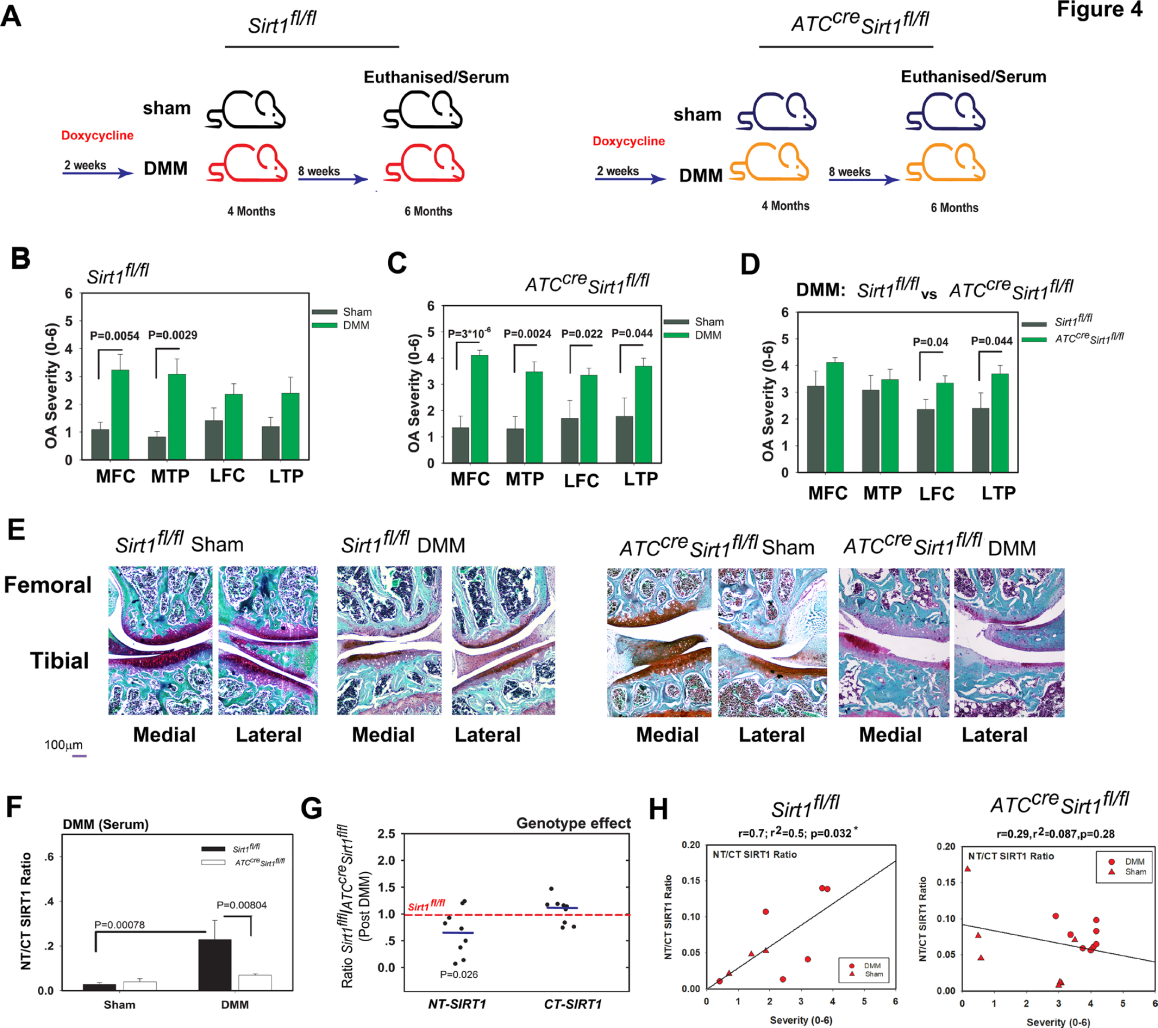
Genetic ablation of *Sirt1* in cartilage reduces the N-terminal (NT)/C-terminal (CT) SIRT1 serum ratio post DMM. (A) Scheme of the experimental set-up: 4-months-old *Sirt1^fl/fl^* and *ATC^cre^Sirt1^fl/fl^* mice (C57BL/6 background) were treated as indicated in the Materials and methods section. They were euthanised 8 weeks post DMM and serum was collected for indirect ELISA analysis to examine NT/CT SIRT1 ratio at experimental endpoint (mice were euthanised at 20 weeks of age). Osteoarthritis (OA) severity ranking based on Glasson 2010 criteria was carried out for medial tibial plateau (MTP), lateral tibial plateau (LTP), medial femoral chondyle (MFC), lateral femoral chondyle (LFC) of the following. (B) DMM (n=8; 50% male) versus sham *Sirt1^fl/fl^*(n=7; 43% male). (C) DMM (n=12, 58% male) versus sham *ATC^cre^Sirt1^fl/fl^* (n=7, 43% male). (D) DMM *Sirt1^fl/fl^* vs *ATC^cre^Sirt1^fl/fl^*. (E) Displays representative histological images following Safranin O/Fast green staining for each genotype and treatment. (F) NT/CT SIRT1 ratio was established based on indirect ELISA method for serum of *Sirt1^fl/fl^* and *ATC^cre^Sirt1^fl/fl^* mice subject to sham or DMM procedures. (G) Individually detected levels of NT or CT SIRT1 variants detected in DMM-treated *ATC^cre^Sirt1^fl/fl^* and compared with *Sirt1^fl/fl^* baseline (broken bar). Statistical significance is indicated by an asterisk (*, p<0.05) based on Mann-Whitney analysis. (H) Correlations of NT/CT SIRT1 ratio versus OA severity per individual mouse within the DMM *Sirt1^fl/fl^* (left graphs) and *ATC^cre^Sirt1^fl/fl^* (right graph). Correlations are presented as Pearson’s correlation (r; 1 being the best linear fit and 0 being the weakest fit), linear regression (r^2^) and p value.

To assess if the elevated level of serum SIRT1 fragments detected in humans and mice with OA is cartilage derived, we employed induced PTOA in our cartilage-specific *Sirt1* knockout mouse model and analysed samples 8 weeks after DMM surgery (figure 4A; illustration of experimental set-up). While control mice developed significant OA lesions in the medial compartments following DMM (figure 4B), mutant mice developed OA lesions in all compartments of the knee (figure 4C). These lesions were more severe in lateral compartments in the mutant than control mice (figure 4D). The subchondral bone thickness and bone volume/tissue volume ratio did not show any significant variations among the genotypes post DMM (online supplementary S6). The analysis of serum showed an expected increase in the NT/CT SIRT1 ratio in control mice after DMM (figure 4F). Interestingly, this increase was not observed in mutant mice, which lack *Sirt1* expression in chondrocytes. The analysis of the serum levels of SIRT1 NT and CT showed that mutant mice had less SIRT1 NT fragment in the circulation than control mice, but similar levels of CT variant (figure 4G). Figure 4H shows significant correlation values in DMM *Sirt1^fl/fl^* (left panel) versus *ATC^Cre^Sirt1^fl/fl^* mice (right panel) when comparing individual NT/CT SIRT1 ratios to OA severity. These data thus demonstrated that the elevated NT/CT SIRT1 ratio in control mice developing PTOA was due to an increased release of NT fragment from chondrocytes into the serum.

**Figure 5:**
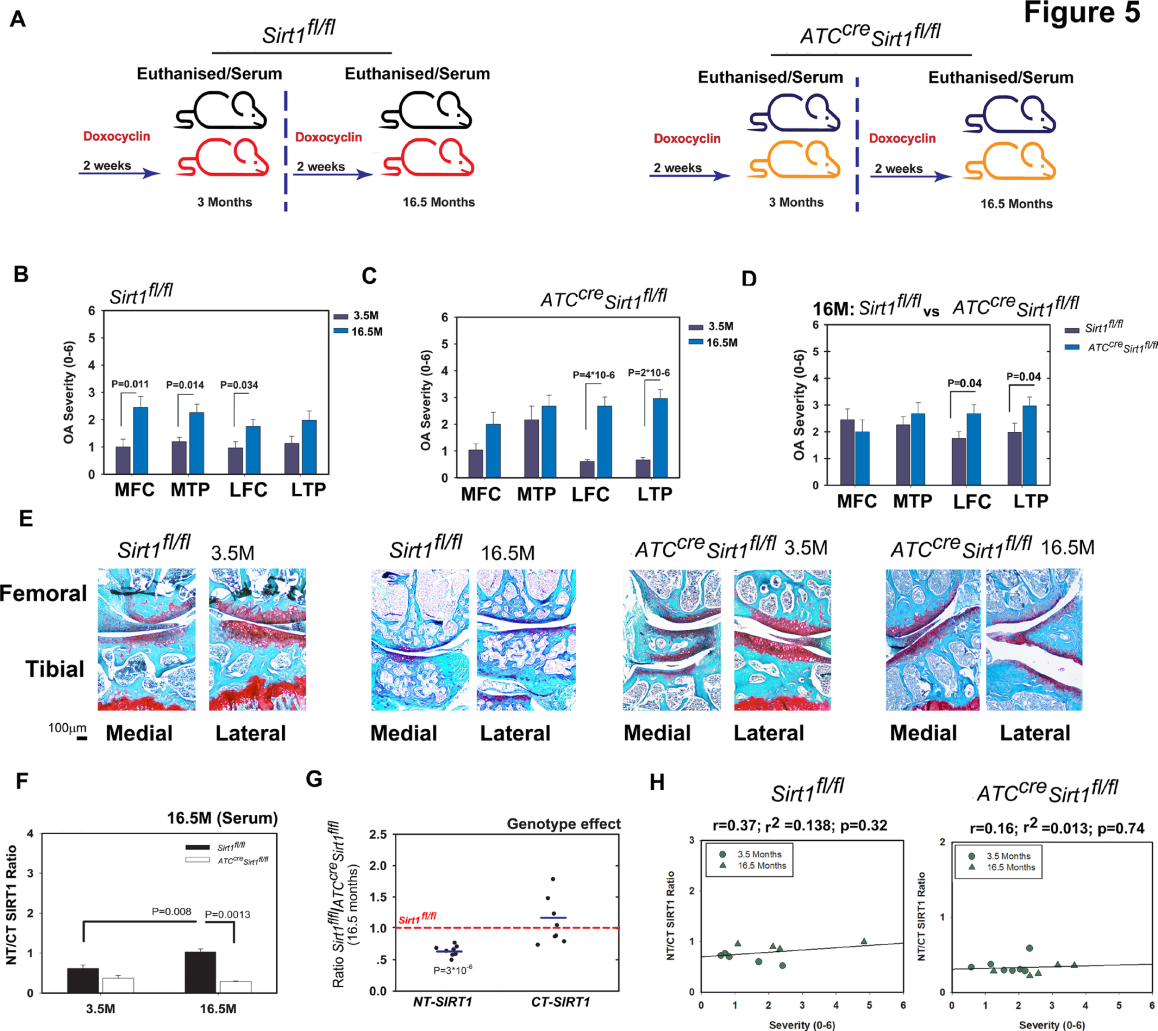
Ablation of Sirt1 in cartilage reduced N-terminal (NT)/C-terminal (CT) SIRT1 ratio in serum of aged versus young mice. (A) Scheme of the experimental set-up: wild-type (WT) *Sirt1^fl/fl^* and *ATC^cre^Sirt1^fl/fl^* mice (C57BL/6 background) were treated as detailed in the Materials and methods section and euthanised at 3.5 and 16.5 months. Following sacrifice, osteoarthritis (OA) severity ranking was carried out based on joint sections stained with Safranin O/Fast green staining and subsequently graded using Glasson 2010 criteria for medial tibial plateau (MTP), lateral tibial plateau (LTP), medial femoral chondyle (MFC) and lateral femoral chondyle (LFC) of the following. (B) 3.5 (n=5, 40% male) versus 16.5 (n=7, 0% male) months Sirt1^fl/+^. (C) 3.5 (n=5, 60% male) versus 16.5 (n=13, 100% male) months *ATC^cre^Sirt1^fl/fl^* and (D) 16.5 months *Sirt1^fl/fl^* versus 16.5 months *ATC^cre^Sirt1^fl/fl^.* (E) Representative histological images (Safranin O/Fast green staining) for each genotype and treatment. (F) NT/CT SIRT1 ratio was established based on the indirect ELISA method for serum of *Sirt1^fl/fl^* and *ATC^cre^Sirt1^fl/fl^* mice subject to sham or DMM procedures. (G) Individually detected levels of NT Sirt1 or CT Sirt1 variants for old (16.5 months) *ATC^cre^Sirt1^fl/fl^* and compared with *Sirt1^fl/fl^* baseline (broken bar). Statistical significance is indicated by an asterisk (*, p<0.05) based on Mann-Whitney analysis. (H) Correlations of NT/CT SIRT1 ratio versus OA severity per individual mouse within the 3.5 months and 16.5 months *Sirt1^fl/fl^* (left graphs) and age-matched *ATC^cre^Sirt1^fl/fl^* (right graph). Correlations are presented as Pearson’s correlation (r; 1 being the best linear fit and 0 being the weakest fit), linear regression (r^2^) and p value.

Next, we compared 3.5-month-old and 16.5-month-old control and mutant mice for age-related OA development (figure 5A; illustration of experimental set-up). Both types of mice developed knee OA on ageing (figure 5B, C, respectively). Their degree of OA severity was similar, except for the lateral compartment, which was more affected in the old mutant than control mice (figure 5D). Analysis of serum showed an expected increase in NT/CT SIRT1 values in old control mice, which was not observed in old mutant mice (figure 5F). This increase was due to a significantly lower level of NT variant in the mutants rather unchanged level of CT variant (figure 5G). Figure 5H shows higher correlation values in 3.5-month and 16.5-month *Sirt1^fl/fl^* mice (left panel) versus age-matched *ATC^Cre^Sirt1^fl/fl^* mice (right panel) when comparing individual NT/CT SIRT1 ratios to OA severity. Thus, like the PTOA mouse model, the ageing mouse model indicated that the elevated NT/CT SIRT1 fragments detected in the serum of mice with OA are derived from chondrocytes.

### The serum NT/CT SIRT1 ratio reflects local clearance of senescent cells

**Figure 6:**
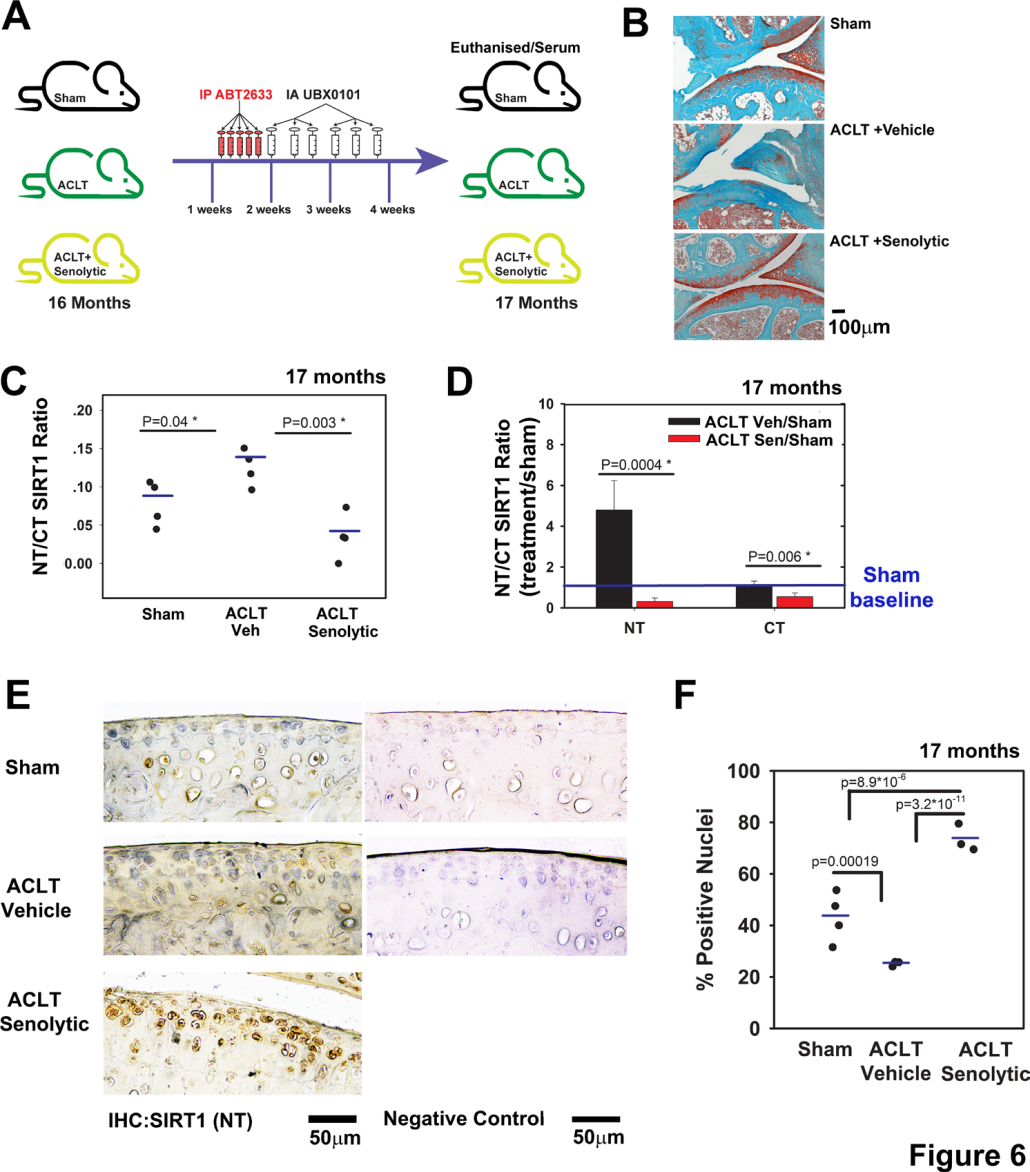
Post-traumatic ACLT-induced osteoarthritis (OA) augments the serum SIRT1 N-terminal (NT)/C-terminal (CT) Sirt1 which were reduced following intra-articular (IA) senolytic administration. (A) Scheme of the experimental set-up; C57B6 mice at age 16 months were subjected to ACLT procedure and treated 7 days post ACLT with 5 consecutive intraperitoneal injections of ABT263, followed by 6 IA injections every other day until sacrifice 28 days post surgery. (B) Representative images of joints stained with Safranin O/Fast green from A (n=4 for each group, ×10 magnification). (C) NT/CT SIRT1 ratio was established based on the indirect ELISA method for serum of mice in A. (D) Individual levels of NT or CT for mice in A (n=4) normalised to the sham mice. (E) Immunohistochemistry for NT-SIRT1 of ACLT-senolytic treated mice (n=3), ACLT-vehicle treated mice (n=3) and sham mice (n=4). (F) Quantification of SIRT1-positive nuclei from total cell population per field, in immunohistochemical sections.

We next sought to examine if combined systemic and intra-articular administration of a senolytic drug would affect these outcomes. Such a treatment was proven efficient in clearing senescent chondrocytes and in limiting PTOA progression of 3-month mice.^5^ Here, we used aged mice (16 months) and subjected them to anterior cruciate ligament transection (ACLT) procedure (figure 6A; illustration of experimental set-up). Similar to previous data with 3-month mice,^5^ representative depictions of histopathology show that vehicle-treated ACLT mice developed OA within 4 weeks (figure 6B), while systemic and local administration of ABT263 and UBX0101, respectively, exhibited OA severity levels comparable to sham surgery (currently under review). In fact, OA severity was significantly increased (threefold) in vehicle-treated ACLT mice versus sham controls (unshown), based on Glasson *et al* grading method.^30^ Moreover, senolytic-treated mice and sham controls showed similar OA severities, which were found to be statistically insignificant (unshown). Importantly, NT/CT SIRT1 ratio was significantly increased in vehicle-treated ACLT mice, as previously observed in the DMM model (figures 2 and 4). Interestingly, ACLT mice treated with the senolytic drug had a lower NT/CT SIRT1 ratio, which correlated with reduced OA severity (figure 6C). The serum levels of both NT and CT dropped after senolytic treatment, with a sharper drop (10-fold) for NT than for CT (twofold; figure 6D) values.

Immunohistochemistry of cartilage tissue sections showed a twofold reduction of NT-SIRT1 staining in vehicle-treated ACTL versus sham mice (figure 6E, F). Senolytic clearance resulted in an increase in the cellular NT-SIRT1 levels compared with vehicle, a trend similar to that observed in younger mice (3.5 months; online supplementary S7A). To further assess the link between senescence and Sirt1, we examined *ATC^Cre^Sirt1^fl/fl^* and *Sirt1^fl/fl^* post*-*DMM (online supplementary S7B, C). Results show that DMM contributed to the appearance of p16^INK4a^ (online supplementary S7B) in articular chondrocytes, versus sham controls. When comparing post-DMM articular cartilage from *Sirt1^fl/fl^ to ATC^Cre^Sirt1^fl/f^* ^l^, it appeared that p16^INK4a^ positive chondrocytes were more abundant in *ATC^Cre^Sirt1^fl/fl^* mice (online supplementary S7B), supporting the concept that lack of SIRT1 promotes senescence, in line with other reports.^31 32^ The data also confirm that administration of senolytic drugs effectively cleared senescent chondrocytes, given that SIRT1 was detected in cells only post treatment (figure 6E, F).

In line with previous observations, the data support that the NT SIRT1 variants detected in serum are derived from damaged cartilage, since their levels were increased in serum and simultaneously reduced in cartilage during ACLT. However, ACLT with senolytic treatment reduced the serum NT/CT SIRT1 ratio and increased the cellular SIRT1 level in articular cartilage. The data support that post traumatic insults induced chondrosenescence and reduced SIRT1 levels in chondrocytes, possibly due to local proinflammatory signals derived from senescent cells releasing SASP vesicles, which have been shown to contain a host proinflammatory mediator, chemokines (interleukin (IL) 1α, IL6, CXCL-8),^33 34^ that elicit tissue damage.^35 36^ While we did not test the presence of SASP vesicles in our report, we envisage that prolonged exposure to SASP may trigger apoptosis of non-senescent cells, resulting in fewer SIRT1-positive cells in articular cartilage. This consequents in increased NT/CT SIRT1 ratio in the circulation.

### Long-term exposure of chondrocytes to proinflammatory cytokines causes cell apoptosis and the release of soluble SIRT1 fragments

To further understand the cellular dynamics leading to the increase in the NT/CT SIRT1 ratio in the serum of patients with OA, we cultured human chondrocytes, stimulated them for 2 or 8 days with tumour necrosis factor alpha (TNFα) and interleukin 1β (IL1β), and assessed cell apoptosis and the NT/CT SIRT1 ratio in conditioned medium. The rate of cell apoptosis was fourfold higher at day 8 than day 2 exposure and this result coincided with a threefold increase in the NT/CT SIRT1 ratio (online supplementary S8A, B). Cell lysates revealed a significantly higher level of 75SIRT1 in cells stimulated with the cytokines for 2 days but not in cells treated for 8 days (online supplementary S8C). These data suggest that the increased rate of cell apoptosis observed during 8 days of cytokine insult resulted in the release of 75SIRT1 to conditioned medium (online supplementary S8B).

**Figure 7:**
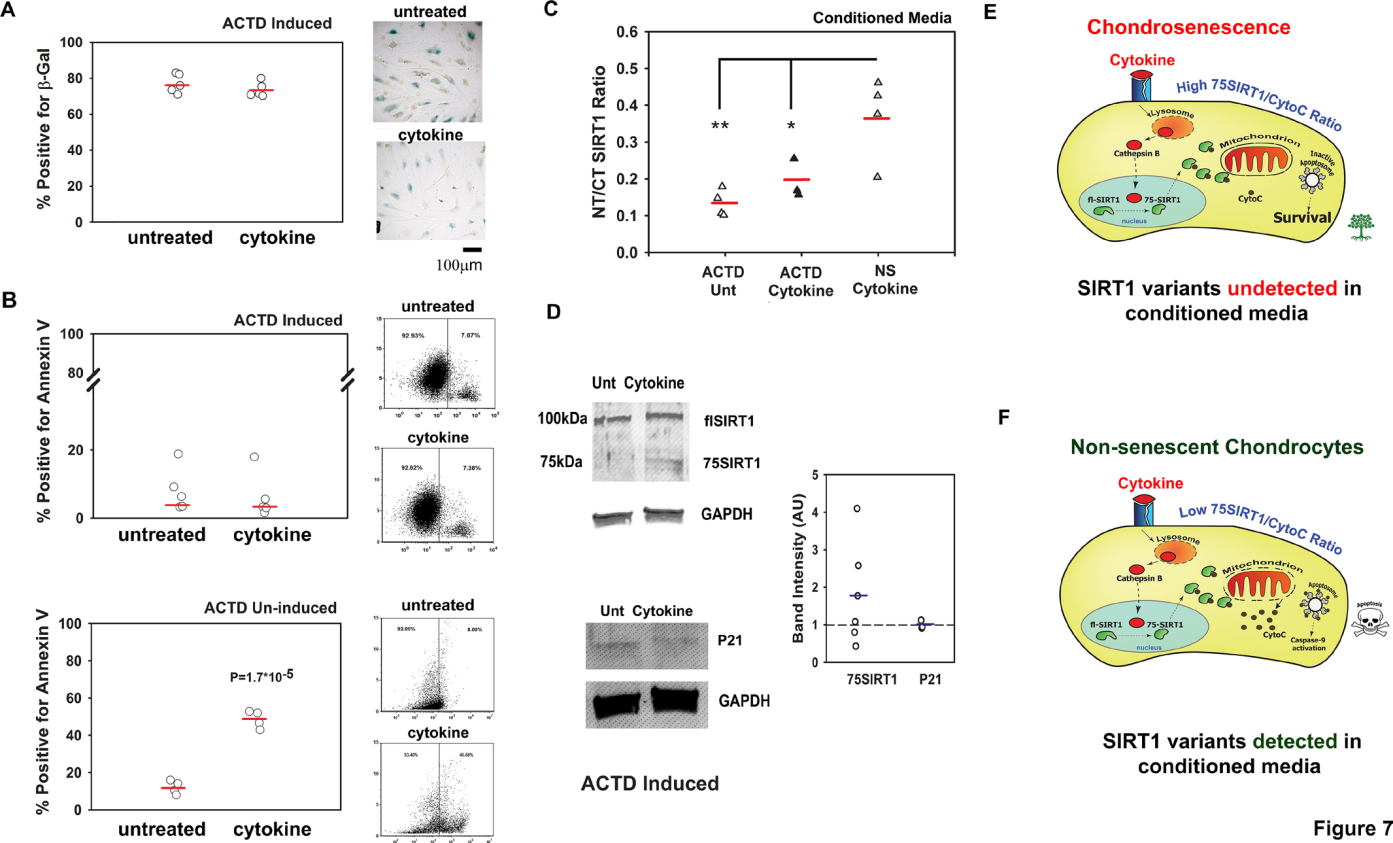
Non-senescent (NS) apoptotic chondrocytes contribute to N-terminal (NT)/C-terminal (CT) SIRT1 ratio in conditioned media. Human chondrocytes were induced to undergo senescence via actinomycin D (ACTD) and thereafter treated for 8 days with cytokines (50 ng/mL tumour necrosis factor alpha and 5 ng/mL interleukin 1β, denoted as ‘cytokine’). (A) Quantification of β-galactosidase positive chondrocytes (n=5). (B) FACS analysis for annexin V positive cells for ACTD-induced cells treated with cytokines (n=5, upper graphs) and ACTD-uninduced cells treated with cytokines for 8 days (n=4, lower graphs). (C) Indirect ELISA for NT/CT SIRT1 in conditioned media derived from ACTD-pretreated chondrocytes (with or without cytokine stimulation) and ACTD-untreated NS chondrocytes stimulated with cytokines for 8 days (n=4). *p<0.05; **p<0.001 for differences of ACTD cytokine (black triangles) treated and untreated (white triangles) conditioned media versus cytokine stimulated conditioned media from chondrocytes uninduced with ACTD (grey triangles; that is, non-senescent or ‘NS’).(D) Immunoblots for NT Sirt1 and P21 for cells in A (n=3). Statistical significance was determined for p<0.05 via Mann and Whitney test. (E) High 75SIRT1/CytoC cellular ratio in chondrosenescence leading to survival under prolonged inflammatory insult. Under such a scenario, SIRT1 variants will not be detected in conditioned media. (F) Low 75SIRT1/CytoC cellular ratio in NS chondrocytes leading to apoptosis under prolonged inflammatory insult (ie, cytokine). Under such a scenario, SIRT1 variants will be detected in conditioned media.

Since senescent chondrocytes are often capable of surviving harsh stress conditions, we postulated that by inducing chondrosenescence, cell death would be impaired and would consequent in lower levels of NT/CT SIRT1 ratio, during prolonged proinflammatory exposure. We therefore induced chondrosenescence with ACTD, as previously described, and confirmed cell senescence using a β-galactosidase staining assay (online supplementary S8D). Interestingly, the numbers of β-galactosidase-positive cells were similar in cytokine-treated and untreated cells (figure 7A). In line with these observations, the percentages of annexin V-positive cells, as measured by fluorescence-activated cell sorting, did not rise on cytokine treatment (figure 7B, upper graph), whereas they increased fourfold for non-senescent chondrocytes (ie, uninduced by ACTD; lower graph of figure 7B). Similarly, conditioned media did not show differences in NT/CT SIRT1 ratio between cytokine-treated and untreated senescent chondrocytes (figure 7C), while non-senescent cytokine-treated chondrocytes showed a fourfold increase in apoptosis and a significant increase in NT/CT SIRT1 ratio in conditioned media. Analysis of adherent chondrocytes by immunoblotting showed similar levels of cyclin-dependent kinase inhibitor 1 (P21 level) confirming that ACTD rendered cell cycle arrest in both cytokine treated and untreated chondrocytes (figure 7D), in consistence with β-galactosidase staining (figure 7A). Moreover, chondrosenscent cells did not exhibit significant changes in intracellular levels of 75SIRT1 following cytokine treatment (figure 7D). These data indicate that senescent chondrocytes may not be affected by cytokine insult and less likely to exhibit lysosomal permeability compared to non-senescent chondrocytes exposed to similar conditions.

The illustration in figure 7E displays a senescent chondrocyte wherein the inflammatory insult will induce Sirt1 cleavage (ie, 75SIRT1 generation), yet given that the mitochondria are impermeable with limited cytochrome C (CytoC) release (ie, high cellular 75SIRT1/CytoC ratio), the cell will remain viable; hence, SIRT1 variants are undetected in surrounding media (figure 7E). On the other hand (figure 7F), non-senescent chondrocytes, subject to prolonged proinflammatory exposure, will generate 75SIRT1 and continuously release CytoC within the cytoplasm (ie, low cellular 75SIRT1/CytoC). Excessive CytoC levels will allow apoptosome assembly, and enhance apoptosis (online supplementary S8.^21^ Apoptotic chondrocytes (mostly non-senescent) will release their content, enabling the detection of SIRT1 variants in surrounding media. Finally, we argue that senescent chondrocytes are often fewer than non-senescent chondrocytes in cartilage; hence, their elimination via senolytic agents will not cause an increase in circulating SIRT1 variants, in line with our observations in figure 6. This highlights the potential value of SIRT1 and its fragments as biomarkers suitable for screening for novel senolytics.

## Discussion

Recent reports have indicated that SIRT1 can be detected in the serum of patients with various pathologies, such as fatty liver disease,^37^ obesity,^18^ chronic obstructive pulmonary disease (COPD)^38^ and Alzheimer’s disease.^39^ Specifically, the serum level of SIRT1 declines in patients suffering from frailty (sarcopenia; cognitive decline; abnormal immune function and neuroendocrine systems; and poor energy regulation,^40^), COPD, metabolic instability and Alzheimer’s disease. These data are rather surprising since SIRT1 is an intracellular deacetylase and it is mainly nuclear.

Despite accumulating data on SIRT1 in the circulation, our study is the first one to analyse the levels of SIRT1 variants in serum. Given that two truncation events have been reported for SIRT1, such an assay may enable better distinction between various conditions, such as obesity and inflammatory conditions.^20 25^ While NT truncation via caspase 1 was correlated with metabolic syndrome and adipose inflammation,^25^ CT via cathepsin B was associated with joint inflammation, as well as other acute inflammatory conditions.^20 27 28 41^ Therefore, we opted to examine the NT/CT SIRT1 ratio, assuming that an increase in this ratio could indicate a local inflammatory response in joint cartilage, due to the presence of IL1β and TNFα, which have both been implicated to promote SIRT1 cleavage in cartilage.^20 21^ Notably, variants resulting from both NT and CT truncation events could not be detected in our ELISA assay, since such variants would lack the domains detected by the antibodies employed.

The increase seen in NT/CT SIRT1 ratio was observed in moderately severe OA resulting from ageing or joint trauma in mouse models and human samples. Moreover, local administration of a senolytic drug reduced the serum NT/CT SIRT1 ratio, indicating that there is a link between cell senescence and gradual loss of SIRT1 in cartilage during OA. These data strongly suggest that the serum NT/CT SIRT1 ratio may be an effective marker of disease severity as well as senolytic drug efficacy. While this biomarker is largely investigative in this report with a small human cohort, the results here support that it may serve as a clinical biomarker for OA-related ‘Burden of Disease’ and/or ‘Efficacy of Intervention’ at least for senolytic drugs, in accordance to BIPED criteria.^8^

Given that SIRT1 is ubiquitously expressed and cleaved in cells present in other inflamed tissues, such as endothelial cells, macrophages and lymph node cells,^26–28^ it was important to assess if indeed serum fragments were cartilage derived. Using a transgenic model, with specific inactivation of *Sirt1* in cartilage, we were able to reduce the increase in the serum NT/CT SIRT1 ratio associated with post-traumatic or age-induced OA. To further understand the mechanism by which SIRT1 fragments emerge in serum, we assessed conditioned media and apoptotic rates of cytokine-exposed chondrocytes. Interestingly, chondrocytes challenged with cytokines underwent increased apoptosis, which was accompanied with an increase in the NT/CT SIRT1 ratio in conditioned medium. On the other hand, chondrocytes undergoing senescence and subsequently exposed to long-term cytokine insult did not show any changes in apoptotic rates or soluble NT/CT SIRT1 ratio. These data are consistent with the proinflammatory effect bestowed by SASP vesicles released from senescent cells.^33–35^ SASP particles released by senescent cells can induce ECM degradation and cell death and can attract immune cells and promote metastasis.^35 36^ SASP-secreting senescent cells bestow a ‘bystander effect’ onto neighbouring cells,^42 43^ creating a harsh environment for non-senescent cells.^34 44 45^

Mechanistically, we speculate that the rise in serum NT/CT SIRT1 ratio is closely correlated with early proinflammatory events in the joint since this stage is also accompanied with a rise in p16^INK4A^-positive cells following joint trauma.^5^ Increased chondrosenescence gradually contributes to a loss of non-senescent chondrocytes, which results in rising levels of the serum NT/CT SIRT1 ratio. Notably, this is a direct response to prolonged inflammatory stress wherein accumulated 75SIRT1 is incapable of sufficiently binding all released CytoC to prevent cell death, under these harsh proinflammatory tissue conditions.^21^ Moreover, given that senescent cells do not show a significant increase in 75SIRT1 following inflammatory stimuli may indicate that their lysosomes are less leaky than in non-senescent cells under the same conditions^46^ and similar to mitochondria of senescent cells.^47^ Under these conditions, it is not surprising that local administration of senolytic drugs to eliminate surviving senescent cells from cartilage ultimately reduced the serum NT/CT SIRT1 ratio which was likely stemming from non-senescent by standard chondrocytes. While these data must be further validated with a clinical grade method and using larger cohorts of patients, we here provide preliminary supporting evidence that the serum NT/CT SIRT1 ratio is indicative of early-stage OA and senolytic drug efficacy.
