# Supplementary material for: Serum NT/CT SIRT1 ratio reflects early osteoarthritis and chondrosenescence
Source: Ann Rheum Dis. 2020 Jul 14;79(10):1370–80. doi: 10.1136/annrheumdis-2020-217072 (PMC7509530; doi:10.1136/annrheumdis-2020-217072)
Supplement: Supplementary data [file annrheumdis-2020-217072supp001.pdf]

***Supplemental Information**: Batshon et al., 2020*

S1: Supplemental text

MATERIALS AND METHODS

Mice related procedures:

All procedures involving mice were carried out in accordance with the guidelines of the ARAC (National Institutes of Health Animal Research Advisory Committee) and AAALAC (Association for Assessment and Accreditation of Laboratory Animal Care International). The Hebrew University Institutional Animal Care and Use Committee approved the study's protocols (MD-12-13383-4; MD-14-14172-2; 18-15660-1). Mice were subjected to 12-h light/dark cycles and received food and water ad libitum. *Sirt1* heterozygous-null mice (129-Sirt1^{tm1Mcby}) were purchased from the RIKEN BioResource Center (Japan). The line was maintained by breeding 129-Sirt1^{tm1Mcby} mice with CD1 wild-type mice. Heterozygous offspring were inbred to generate *Sirt1* wild type (WT) with a highly enriched CD1 background. WT male and female mice were euthanized at 3 months (3M) and 15M (n>5), for age-dependent OA (ADOA), while 3M WT mice were subjected to surgical destabilization of the medial meniscus (DMM) or a sham procedure, as previously described [1]. Mice were euthanized by cervical dislocation following anesthesia (7mg/kg ketamine and xylazine 200 mg/kg). Serum was collected under anesthesia from the tail vein every 2 weeks post-DMM and from the orbital sinus at the end of the experiments.

C57BL/6 mice (Jackson lab) were subjected at 3M or 16M of age to anterior cruciate ligament transection (ACLT) [2] and were euthanized 1 month later for serum analysis and grading of OA severity based on OARSI guidelines [3]. As previously described, 3M mice were injected 2 weeks post-

Batshon G, *et al. Ann Rheum Dis* 2020; 79:1370–1380. doi: 10.1136/annrheumdis-2020-217072

***Supplemental Information**: Batshon et al., 2020*

ACLT, with 1 mM UBX0101 (6 intra-articular injections, once every 2 days over the next 2 weeks), until sacrifice 28 days post ACLT [4]. Alternatively, 16M mice were subject to ACLT or sham procedures and treated 7 days after the procedure with systemic injections of ABT263 (100μg in saline/kg weight; IP) for 5 consecutive days. 14-days after the ACLT procedure, the mice were treated with UBX0101 every other day with 6 intra-articular injections (10μL; 1 mM UBX0101 in saline). Mice were sacrificed 28 days after ACLT or sham. UBX0101 (Unity Biotechnology, CA) is a small molecule acting as a potent senolytic inhibitor, which disrupts MDM2/p53 protein interaction, and thereby eliminates senescent cells. Similarly, ABT263 disrupts Bcl2 in senescent cells and previously reported to eliminate senescent cells [5].

Cartilage-specific Sirt1 knockout mice were generated on a C57BL/6 background using ATC^{Cre} mice [6] and Sirt1$^{fl/fl}$ mice (Jackson Laboratory, #008041). Pregnant dams were given drinking water supplemented with doxycycline (0.8 mg/400 mL; Biobasic, Canada, cat# 24390-14-5) during the gestational days 11.5 to E17.5 to assess differences in skeletal development and costal chondrocyte cultures between Sirt1$^{fl/fl}$ (control) and ATC^{Cre}Sirt1$^{fl/fl}$ (mutant) littermates, based on previous protocols [7, 8]. Adult Sirt1$^{fl/fl}$ and ATC^{Cre}Sirt1$^{fl/fl}$ mice were provided doxycycline in drinking water (0.8 mg/400 ml) for 2 weeks before euthanasia and from 2 weeks before DMM. DMM and sham mice were euthanized 8 weeks post-surgery. For aging experiments, mice were euthanized at 3.5M and 16.5M. Serum was collected as indicated above.

Batshon G, *et al. Ann Rheum Dis* 2020; 79:1370–1380. doi: 10.1136/annrheumdis-2020-217072

***Supplemental Information**: Batshon et al., 2020*

Human cell culture and serum collection

All procedures were performed with Hadassah Medical Center Institutional Review Board approval, in accordance with the Helsinki Declaration of ethical principles for medical research involving human subjects. Following written informed consent, articular cartilage was obtained from OA patients at the time of total knee arthroplasty (n=25, mean age 71 years, mean body mass index 30.5 kg/m^2, Kellgren and Lawrence score 3-4). Intact and degenerated tissues were isolated as described [9]. For cell culture, cartilage was dissected, chondrocytes were isolated and plated as described [10]. Unless otherwise indicated, all cell culture reagents were purchased from Biological Industries (Beit-Haemek Kibutz, Israel). Cells were plated in the amount of 1.5 x 10^6 per 140-cm^2 tissue culture dish and were grown to confluence under standard conditions (37°C, 5% CO_2) in DMEM medium (Sigma-Aldrich, St Louis, MI) with 10% FCS, 1% penicillin-streptomycin, and 1% Amphotericin B (03-033-1B). All experiments were conducted on chondrocytes at the passages 0 to 3. Cells were treated with the pro-inflammatory cytokines IL1β (5 ng/mL) and TNFα (50 ng/mL) in defined serum-free medium (BIO-MPM 1). Conditioned media were filtered with a 10-kDa cutoff Amicon filter (Millipore, MA) and processed for enzyme-linked immunosorbent assay (ELISA) as described below.

To induce senescence, chondrocytes were treated with 10 µg/ml actinomycin D (ACTD) in growth medium [11] for 24 h. Next, the medium was renewed, and cells cultured for 10 days prior to cytokine treatment. Senescence was detected with a ß-galactosidase activity colorimetric assay in chondrocytes plated on glass coverslips, resulting in blue-stained cells.

Batshon G, *et al. Ann Rheum Dis* 2020; 79:1370–1380. doi: 10.1136/annrheumdis-2020-217072

***Supplemental Information**: Batshon et al., 2020*

Briefly, following culture and treatment, chondrocytes were washed with PBS (pH 7.4, room temperature), and fixed with 0.2% Glutaraldehyde (Sigma-Aldrich, St Louis). Thereafter, cells were incubated with X-Gal solution (Inalco, San Luis Obispo, CA) at 37°C for 2 h and washed with PBS. Stained cells were visualized using a Nikon Eclipse Ci Y-TV55 upright light microscope and quantified visually (5-10 fields per coverslip).

Human serum and synovial fluid (n = 28) were obtained after approval by the Ethical Review Committee of the Faculty of Medicine (Justus-Liebig-University Giessen, Germany), and all donors provided written informed consent to donor samples for research. Serum and synovial fluid (SF) samples from patients with late OA (n = 9, av. age 67.1, av. BMI 29.2, 60% male) were collected with sterile needles and syringes before joint replacement surgery or arthroscopic meniscal and/or cartilage repair performed at the Department of Orthopaedics and Orthopaedic Surgery, University Hospital Giessen (UKGM, Giessen, Germany). Serum and SF from patients with early stage OA (n = 9, av. age 47.2, av. BMI 27.7, 70% male) were obtained before arthroscopic repair of cartilage and/or injured menisci or ruptured cruciate ligaments or synovectomy at the same university clinic. OA severity was evaluated in patients based on the macroscopic appearance during arthroscopy or surgery of 6 cartilage surfaces—the patella, trochlea, medial and lateral femur and tibia—using the Outerbridge scale, as previously described [12]. Joints with an average Outerbridge score of ≤2 were classified as having early OA, and joints with an average Outerbridge score of ≥2 were classified as having late OA [13].

Batshon G, *et al. Ann Rheum Dis* 2020; 79:1370–1380. doi: 10.1136/annrheumdis-2020-217072

***Supplemental Information**: Batshon et al., 2020*

As non-OA controls, sera samples were obtained from 10 healthy donors (av. age 22.5, BMI 22.6, 40% male), with no documented history of joint disease. Synovial fluid (SF) was taken from a separate group of cadavers (n=11; 81% males; average age, 23.1; average BMI, 22.6). The control cohort was considered joint healthy due to the selected young adult age and exclusion criteria applied, e.g. any joint diseases. The applied inclusion and exclusion criteria of recruited donors were already described earlier [14].

Polymerase chain reaction and mouse genotyping

mRNA was isolated using RNeasy columns (Qiagen, Germany) and cDNA was prepared using OneStep RT-PCR kit (Invitrogen, CA) as previously described [7, 12, 13]. PCR primers were used for *Col2a1* (F, GAGCCCTGCCGGATCTGT; R, GAGGCAGTC TTTCACGTCTTC), *Mmp13* (F, AGTTTGCAGAGCGCTACCTGAGAT; R, TTTGCCAGTCACCTCTAAGCCGAA), *Gapd*h (F, CAAGGCTGAGAACGGGAAGC; R, AGGGGGCAGAGATGATGACC).
Mouse genotyping was carried out using DNA isolated from tail tips, *Sirt1* Flox primers (F, GGTTGACTTAGGTCTTGTCTG; R, CGTCCCTTGTAATGTTTCCC) and Cre Primers (F, TGAGGTTCGCAAGAACCTGATGGA; R, GCCGCATAACCAGTGAAACAGCAT).

Histology and immunohistochemistry

Mouse and human knee cartilage samples were processed for histology as reported [3,15]. Briefly, samples were fixed for 3 days in 4% formaldehyde and decalcified for 21 days in 10% EDTA, pH=7.5 (replenished every 3 days).

Batshon G, *et al. Ann Rheum Dis* 2020; 79:1370–1380. doi: 10.1136/annrheumdis-2020-217072

***Supplemental Information**: Batshon et al., 2020*

Samples were dehydrated in a graded series of ethanol washes before being embedded in paraffin and sectioned to 7-μm slices. For immunohistochemistry, sections were digested with 1 mg/ml hyaluronidase (Sigma-Aldrich, cat# H3506) in PBS at pH 6 for 1 h at 37˚C and stained with DAB substrate kit (cat# DAB057) after overnight incubation with p16^{INK4a} (cat# ab54210, Abcam, UK), CT-Sirt1 (cat# 2028, Cell Signaling, MA) or NT-Sirt1 (cat# 07-131, Merck-Millipore, MA) primary antibody. ZytoChem Plus HRP polymer anti-rabbit (Zytomed Systems, Germany, cat# ZUC032) was used as a secondary antibody and staining substrate. Negative controls were incubated with secondary antibody alone and counterstained with hematoxylin. For histopathology scoring, joint sections were stained with 0.5% Safranin O (cat# 1.15948, Merck) and 0.1% Fast green (cat# 1.04022, Merck-Millipore, MA), following staining with Weigert's Iron Hematoxylin (Merck-Millipore, Merck kit cat# 1.15973). Stained sections were visualized under light microscopy. OARSI histopathology grading was carried out by at least 3 blinded referees using the Glasson scoring method [16].

Microcomputed tomography (μCT)

Knee joints were fixed in 4% paraformaldehyde solution for 48 h and subsequently stored in 70% EtOH for µCT assessment of calcified tissue using Skyscan 1174 (Bruker, Belgium; 50 kV; 0.25-mm aluminum filter; 4000 ms exposure time; 6.4 µm voxel size; 0.4° rotation angle; 7.95 µm pixel size). Scans were reconstructed using NRecon software (Bruker, Belgium). The area of interest was selected as the tibial subchondral bone plate using CTan (Bruker, Belgium). Compartments were manually segmented into medial

Batshon G, *et al. Ann Rheum Dis* 2020; 79:1370–1380. doi: 10.1136/annrheumdis-2020-217072

subchondral bone plate and lateral subchondral bone plate. 3D-analysis was assessed to quantify subchondral bone plate volume and thickness. 3D-images were produced with Dragonfly software (ORS, Montreal, Canada).

Fluorescence-activated cell sorting (FACS)

Cell apoptosis was assessed by FACS using an MEBCYTO Apoptosis kit (MBL, MA). Briefly, following treatment, adherent human chondrocytes and cell debris were collected by centrifugation (5 min at 1000 g) and pellets were washed twice with PBS. Cells were resuspended at $1x10^5$/mL in binding buffer and stained with 3μl fluorescein isothiocyanate (FITC)-labeled annexin V (Annexin-V) and 5 μl propidium iodide (PI). As positive control, human chondrocytes were treated with 1 μM Etoposide (Sigma-Aldrich, Saint Louis, MO) for 24h to induce apoptosis. Data were collected and analyzed using FACScan or CellQuest software (Becton Dickinson and Co., Franklin Lakes, NJ).

SIRT1 plasmids, protein enrichment and immunoblotting

A pcDNA-Flag-His-SIRT1 expression plasmid for human full-length (fl) SIRT1 was a kind gift of Prof. Danny Rienberg (New York University). Based on this construct, human and mouse cleaved SIRT1 plasmids encoding the 75SIRT1 protein (i.e. a.a.1-534 of SIRT1; approx. 75kDa in molecular weight of the translated protein) were designed [17].

The SIRT1 proteins were expressed in HEK293 cells for 36 h following plasmid transfection with a GenJet™ DNA In Vitro Transfection Reagent (SignaGen Laboratories). Cell lysates were made in 20 mM Tris-HCl, 250 mM NaCl, 10 mM Imidazole, pH 8.0, buffer containing a cocktail of protease

inhibitors and were subjected to three freeze-thaw cycles. Clear supernatants containing the His-tagged proteins were loaded on Ni-NTA columns and the bound proteins were eluted with an imidazole gradient (20 mM Tris-HCl, 250 mM NaCl, 250 mM Imidazole, 10% glycerol, pH 7.2). The eluted fractions were examined for purity using the Bradford assay and using Coomassie blue staining for SDS-PAGE gels. Protein fractions were pooled and concentrated using a 30-kDa cutoff Amicon filter (Merck-Millipore). Mutant proteins (mouse or human 75SIRT1 and flSIRT1) were enriched to generate ELISA standard curves. Enrichment was achieved by mass spectrometry (e.g. average 22-fold enrichment for murine flSIRT1; average 46-fold enrichment for murine 75SIRT1; average 6.9-fold enrichment for human flSIRT1; average 47-fold enrichment for human 75SIRT1).

For western blotting, protein extracts were made in RIPA buffer (25mM Tris-HCl pH 7.4, 150 mM NaCl, 1% NP-40, 1% sodium deoxycholate, 0.1% SDS) containing complete cocktail inhibitors (Roche, Germany). Extracts were run on a standard 10% SDS PAGE gels and finally transferred to PVDF membranes. Anti-NT-SIRT1 (IB-1:4000, Merck-Millipore, Billerica, MA, USA; cat# 07-131), Anti-CT-SIRT1 (cat# 2028, Cell Signaling, MA), P21 (cat# 556431, BD Biosciences, CA), β-actin antibody (cat# SC-47778, Santa Cruz, CA) and GAPDH (cat# CB1001, Merck-Millipore), were used as primary antibodies for western blot analysis, while secondary antibodies were Anti-mouse alkaline-phosphatase (AP)-conjugated (Sigma-Aldrich, St Louis, MO, cat# A3562), Anti-rabbit AP-conjugated (Sigma-Aldrich, St Louis, MO, cat# A3687).

***Supplemental Information**: Batshon et al., 2020*

Enzyme-linked immunosorbent assay (ELISA) to detect SIRT1 variants

Indirect ELISA was carried out for serum samples and compared to standard curves ranging from 0.2 to 100 ng/mL full-length human or mouse SIRT1 protein incubated with a C-terminus reactive antibody (antibodies detailed in Table 1) or 0.2 to 100 ng/mL human 75SIRT1 or mouse flSIRT1 incubated with an N-terminus reactive antibody (antibodies detailed in Table 1).

Diluted serum (1:2000) and standard curves were allowed to adhere to an opaque ELISA 96-well plate overnight. Next day, samples were blocked with PBS solution containing 1.5% BSA and 0.05% Tween 20 (blocking solution) for 3 h, washed three times with PBS-Tween and incubated with primary antibody overnight. Following three washes, the plate was incubated with secondary anti-rabbit HRP antibody (111-035-111, Jackson Laboratories) for 1 h at room temperature and washed five times with PBS-Tween. Substrate signal was read using a SuperSignal ELISA Femto (cat# 37074, Thermo Fischer Scientific, MA, US) kit or TMB (cat# 0410 Southern Biotech, AL, US). Data were analyzed using TECAN infinite M200PRO software. ELISA was validated for percent coefficient of variation (%CV) between plates, Spike in and dilution recovery (**Table 1**). Figure 1 illustrates the ELISA assay employed for analysis of serum and conditioned media. Figure **S2** shows specificity of antibodies to the full length and 75SIRT1 of murine and human origin.

Sandwich ELISA was performed by adsorbing chicken anti-SIRT1 (NT) (Cat#146663, US Biological Life Sciences, MA) to a 96-well opaque (white) ELISA plate (1:1000 in PBS) at 4°C, overnight. Next, serum (1:8 in PBS) or human SIRT1 standard (cat# 7714-DA, R&D systems, MN; standard range

Batshon G, *et al. Ann Rheum Dis* 2020; 79:1370–1380. doi: 10.1136/annrheumdis-2020-217072

1.5-50ng/mL) was added and incubated overnight, 4°C, followed by blocking (PBS solution containing 1.5% BSA and 0.05% Tween 20), for 3 h, and three washes.

Either NT-SIRT1 reactive (Millipore cat #07-131; 1:2000 in blocking solution) or CT-SIRT1 reactive (Bethyl Laboratories cat #A300-688; 1:2000 in blocking solution) were added and incubated 3 h at room temperature. Finally, the plate was incubated with a secondary anti-rabbit HRP antibody (111-035-111, Jackson Laboratories) for 1 h at room temperature, and signal was read using a SuperSignal ELISA Femto (Thermo Fischer Scientific) kit or TMB reagent. Data were analyzed using TECAN infinite M200PRO software.

Statistical analysis

Statistical significance was assumed for confidence levels greater than 95% ($p<0.05$). Mann-and-Whitney and t-tests were carried out to determine the differences between two equivalent treatments within a group ($n \geq 3$). Error bars indicated the standard error or standard deviation around the mean value of data point, as detailed in the figure legends.

Pearson correlation was assessed for human serum samples and mice samples, assuming a confidence level of confidence levels greater than 95% ($p<0.05$), to be significant. Notably, Pearson's correlation (r) that is closer to 1 indicates a good fit to linear regression, while values closer to 0 indicate weak fit to linear regression. Regression (r^2) indicates the variation around the linear regression line.

REFERENCES

***Supplemental Information**: Batshon et al., 2020*

1. Glasson SS, Blanchet TJ, Morris EA. The surgical destabilization of the medial meniscus (DMM) model of osteoarthritis in the 129/SvEv mouse. *Osteoarthritis and cartilage* 2007; 15(9):1061-1069.
2. Lorenz J, Grassel S. Experimental osteoarthritis models in mice. *Methods Mol Biol* 2014; 1194:401-419.
3. Glasson SS, Chambers MG, Van Den Berg WB, et al. The OARSI histopathology initiative - recommendations for histological assessments of osteoarthritis in the mouse. *Osteoarthritis Cartilage* 2010; 18 Suppl 3:S17-23.

.4 Jeon OH, Kim C, Laberge RM, et al. Local clearance of senescent cells attenuates the development of post-traumatic osteoarthritis and creates a pro-regenerative environment .*Nat Med* 2017; 23(6):775-781.

.5 Chang J, Wang Y, Shao L, et al. Clearance of senescent cells by ABT263 rejuvenates aged hematopoietic stem cells in mice. *Nat Med* 2016; 22(1):7.8-83

.6 Dy P, Wang W, Bhattaram P, et al. Sox9 directs hypertrophic maturation and blocks osteoblast differentiation of growth plate chondrocytes. *Dev Cell* 2012; 22(3):597-609.

7. Hilton MJ. Skeletal Development and Repair. Humana Press, Totowa, NJ: 2016.
8. Gosset M, Berenbaum F, Thirion S et al. Primary culture and phenotyping of murine chondrocytes. *Nat Protoc* 2008;3:1253-60.
9. Ben-Aderet L, Merquiol E, Fahham D, et al. Detecting cathepsin activity in human osteoarthritis via activity-based probes. *Arthritis Res Ther* 2015; 17.

10. Derfoul A, Miyoshi AD, Freeman DE, et al. Glucosamine promotes chondrogenic phenotype in both chondrocytes and mesenchymal stem cells and inhibits MMP-13 expression and matrix degradation. *Osteoarthritis Cartilage* 2007; 15(6):646-655.
11. Rose J, Soder S, Skhirtladze C, et al. DNA damage, discoordinated gene expression and cellular senescence in osteoarthritic chondrocytes. *Osteoarthritis and cartilage* 2012; 20(9):1020-1028.
12. Outerbridge RE. The etiology of chondromalacia patellae. *J Bone Joint Surg Br* 1961; 43-B:752-757.
13. Kosinska MK, Liebisch G, Lochnit G, et al. A lipidomic study of phospholipid classes and species in human synovial fluid. *Arthritis Rheum* 2013;65(9):2323-2333.
14. Oppenheimer H, Kumar A, Meir H, et al. Set7/9 Impacts COL2A1 Expression Through Binding and Repression of SirT1 Histone Deacetylation. *J Bone Miner Res* 2014; 29(2):348-360.
15. Elayyan J, Lee EJ, Gabay O, et al. LEF1-mediated MMP13 gene expression is repressed by SIRT1 in human chondrocytes. *FASEB J* 2017; 31(7):3116-3125.
16. Glasson SS, Chambers MG, Van den Berg WB, et al. The OARSI histopathology initiative - recommendations for histological assessments of osteoarthritis in the mouse. *Osteoarthr Cartilage* 2010; 18:S17-S23.
17. Kumar A, Daitsh Y, Ben-Aderet L, et al. A predicted unstructured C-terminal loop domain in SIRT1 is required for cathepsin B cleavage. *J Cell Sci* 2018; 131.(16)

***Supplemental Information**: Batshon et al., 2020*

Figure S2

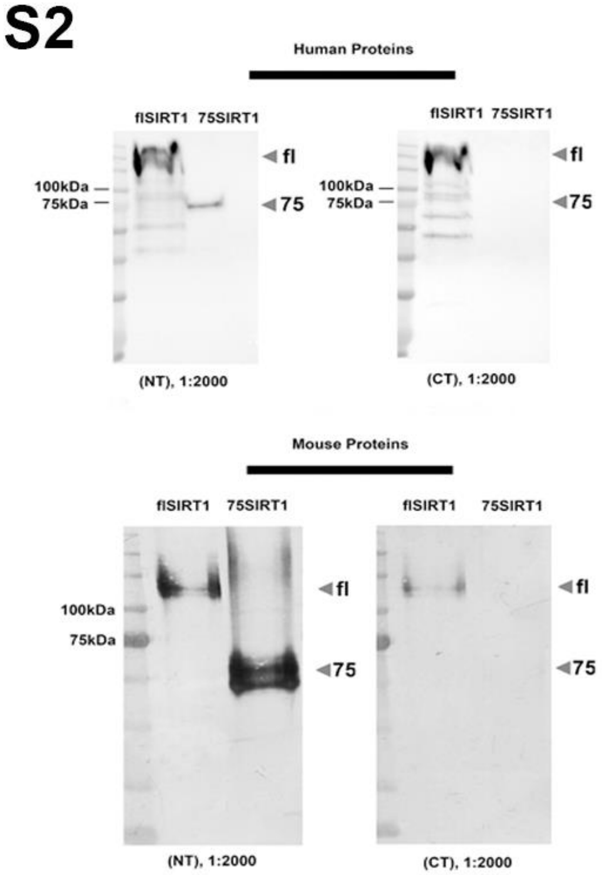

<u>S2</u>: **<u>Antibody reactivity to human and mouse variants</u>**: Human flSIRT1 and 75SIRT1 (10 μg protein) were loaded on a gel and incubated either with an NT-reactive antibody (left upper blot) or CT-reactive antibody a (right upper blot), as detailed in Table 1. Mouse flSIRT1 and 75SIRT1 (5 μg protein) were

loaded on a gel and incubated either with NT-reactive antibody (left bottom blot) or CT-reactive antibody (right bottom blot). Results show that the NT-reactive antibody expectedly recognized both full length (fl) and 75SIRT (human and mouse origin), while CT-reactive antibody recognized only full-length human or mouse SIRT1.

Batshon G, *et al. Ann Rheum Dis* 2020; 79:1370–1380. doi: 10.1136/annrheumdis-2020-217072

***Supplemental Information**: Batshon et al., 2020*

Figure S3

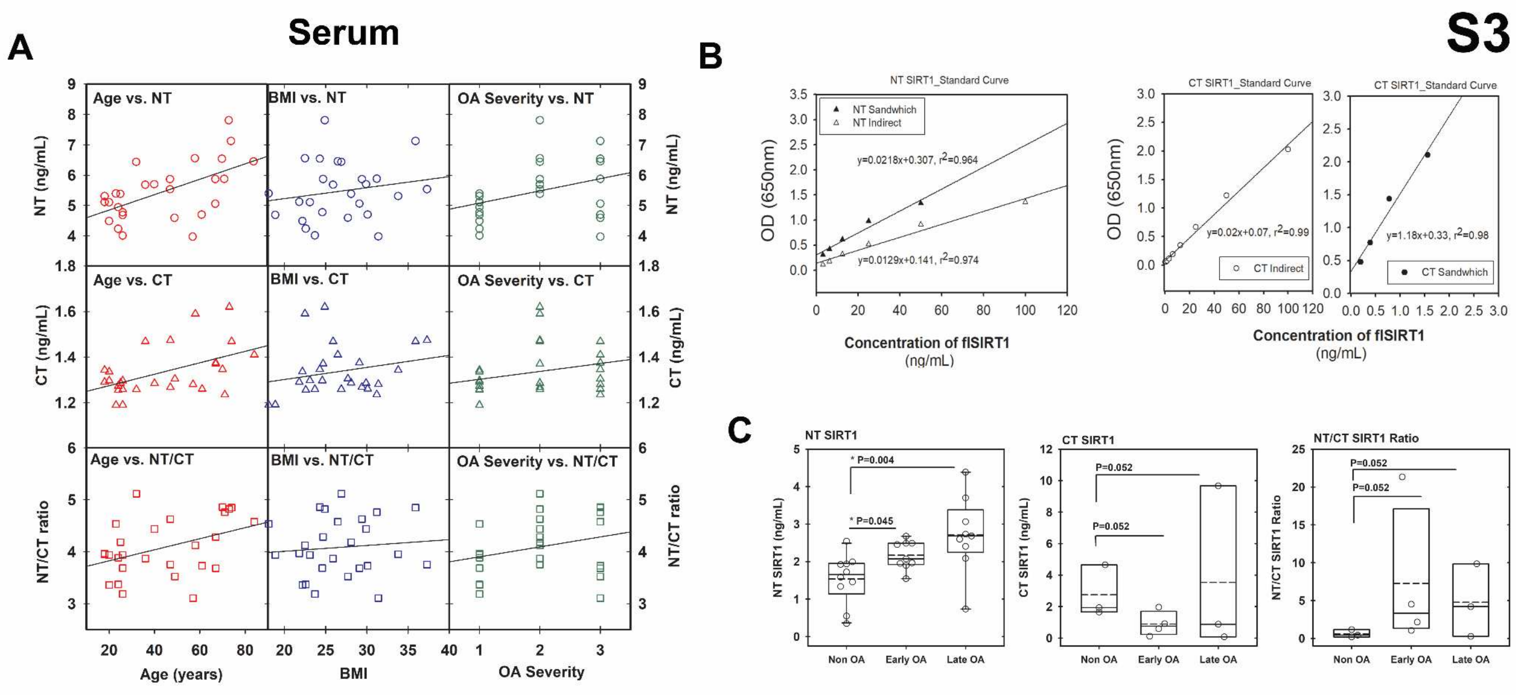

S3:

Pearson's Correlation	NT			CT			NT/CT		
	R	R^2	P	R	R^2	P	R	R^2	P
AGE	0.571	0.326	0.0019*	0.492	0.242	0.0092*	0.409	0.167	0.034*
BMI	0.198	0.039	0.322	0.25	0.063	0.206	0.104	0.0108	0.606
OA SEVERITY	0.353	0.125	0.0705	0.264	0.070	0.182	0.286	0.082	0.147

Indirect ELISA

Pearson's Correlation	NT			CT			NT/CT		
	R	R^2	P	R	R^2	P	R	R^2	P
AGE	0.561	0.315	0.0019*	0.052	0.0028	0.884	0.047	0.0023	0.89
BMI	0.46	0.212	0.013*	0.18	0.032	0.62	0.131	0.0174	0.718
OA SEVERITY	0.571	0.326	0.0015*	0.11	0.012	0.76	0.258	0.066	0.47

Sandwich ELISA

Batshon G, *et al. Ann Rheum Dis* 2020; 79:1370–1380. doi: 10.1136/annrheumdis-2020-217072

***Supplemental Information**: Batshon et al., 2020*

Serum correlations to clinical parameters: Pearson's correlation (r), linear regression (r^2) and p-value for NT, CT (ng/mL) or NT/CT SIRT1 ratio values obtained from serum **(A)** via indirect ELISA method to Age, Body mass index (BMI) and OA severity (1=non-OA; 2=early OA; 3=late OA). Lower table indicates all correlation values. **(B).** Shows indirect and sandwich ELISA standard curve values obtained with pure human Sirt1. **(C**) Analysis of the same human cohorts using a sandwich ELISA platform (Materials and methods), after exclusion of undetected values in CT SIRT1 samples. Graphs represent a median in black bar and average in broken black bar within the box. Whiskers represent first and third quartile sample values. Statistical significance is indicated by an asterisk (*) ($p<0.05$) based on Mann Whitney test. Correlations (table below graph) as in A, are presented by Pearson's correlation (r; 1 being the best linear fit and 0 being the weakest fit), linear regression (r2) and p-value ($p<0.05$ denoted with asterisks in red font).

Batshon G, *et al. Ann Rheum Dis* 2020; 79:1370–1380. doi: 10.1136/annrheumdis-2020-217072

***Supplemental Information**: Batshon et al., 2020*

Figure S4

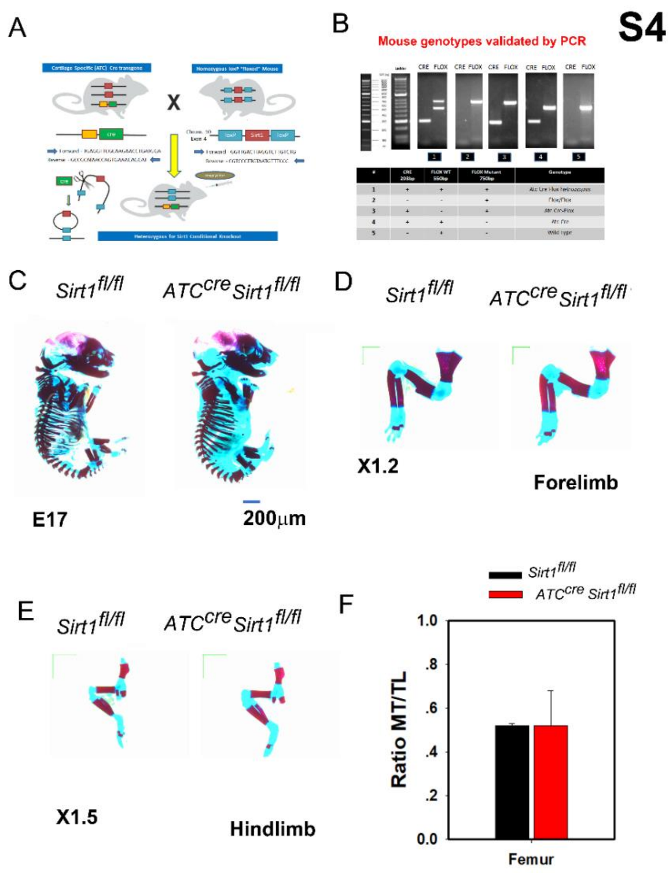

<u>S4:</u> Cartilage specific Sirt1-null mice do not show a growth phenotype. Schematic illustration **A**. showing crossbred Floxed mice (*Sirt1*$^{fl/fl}$) with ATC^{cre} mice to generate *ATC*^{cre}*Sirt1*$^{fl/fl}$ which possess cartilage-specific Sirt1 ablated mice. **B**. PCR genotyping tests generating 750bp fragments upon Flox positive genotype (see lane 1) and 293bp upon ATC positive genotype (see lane 2). The *ATC*^{cre}*Sirt1*$^{fl/fl}$ transgenes (see lane 3) shown for both fragments (750 and 293 bp). **C-E**. Whole skeletal segments (**C**), Forelimb (**D**) and hindlimb **(E**) from E17 littermate embryos were obtained after Doxycycline induction of dams, stained via alizarin red and alcian blue. **F**. captured images

Supplemental Information: Batshon et al., 2020

were quantified for the ratios of Mineralized Tissue (MT) vs Total Length (TL of femur) (n=4).

Figure S5

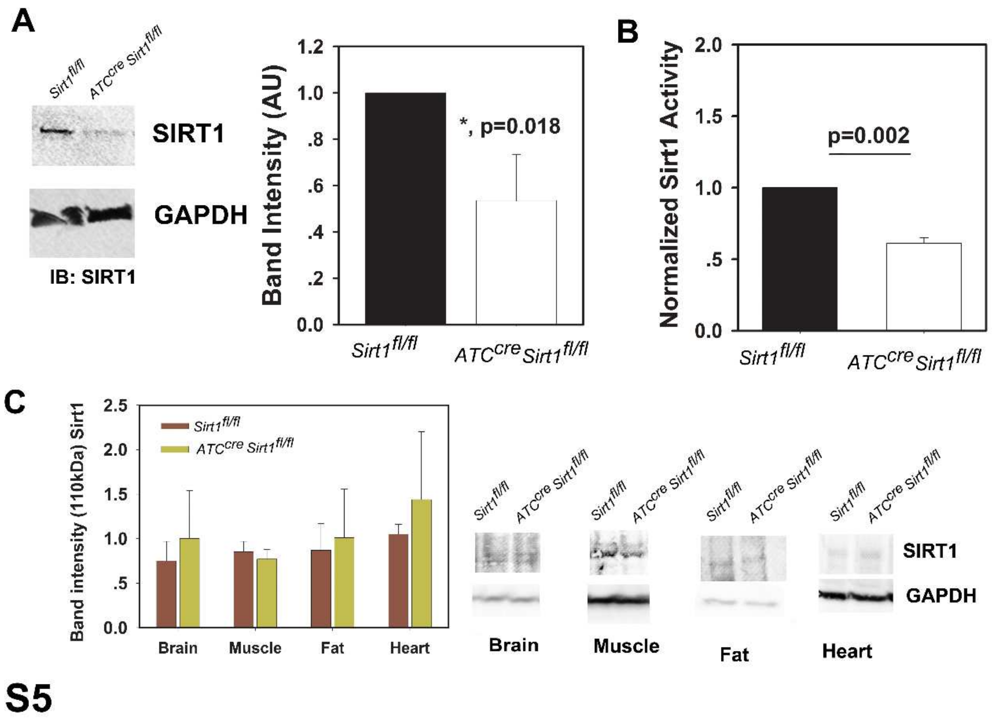

<u>S5:</u> Sirt1 levels and activity are reduced in cartilage-specific Sirt1 knock out mice. (A) Immunoblot from costal cartilage of E17 *Sirt1*$^{fl/fl}$ and *ATC*^{cre}*Sirt1*$^{fl/fl}$ mice (n=4 each). Band intensity was quantified and normalized to GAPDH (left panel). **(B)** Sirt1 activity assay of embryonic costal cartilage of *Sirt1*$^{fl/fl}$ and *ATC*^{cre}*Sirt1*$^{fl/fl}$ mice, showing reduced Sirt1 activity in *ATC*^{cre}*Sirt1*$^{fl/fl}$ (n=4 each). **(C)** Immunoblot (right panel) from protein extract of *Sirt1*$^{fl/fl}$ and *ATC*^{cre}*Sirt1*$^{fl/fl}$ mice organs (brain, muscle, fat, heart), showing that Sirt1 protein levels are unchanged (n=4). Band intensities were quantified and

***Supplemental Information**: Batshon et al., 2020*

normalized to GAPDH (left panel). Mann and Whitney statistical test was applied to determine significance at p<0.05.

Figure S6

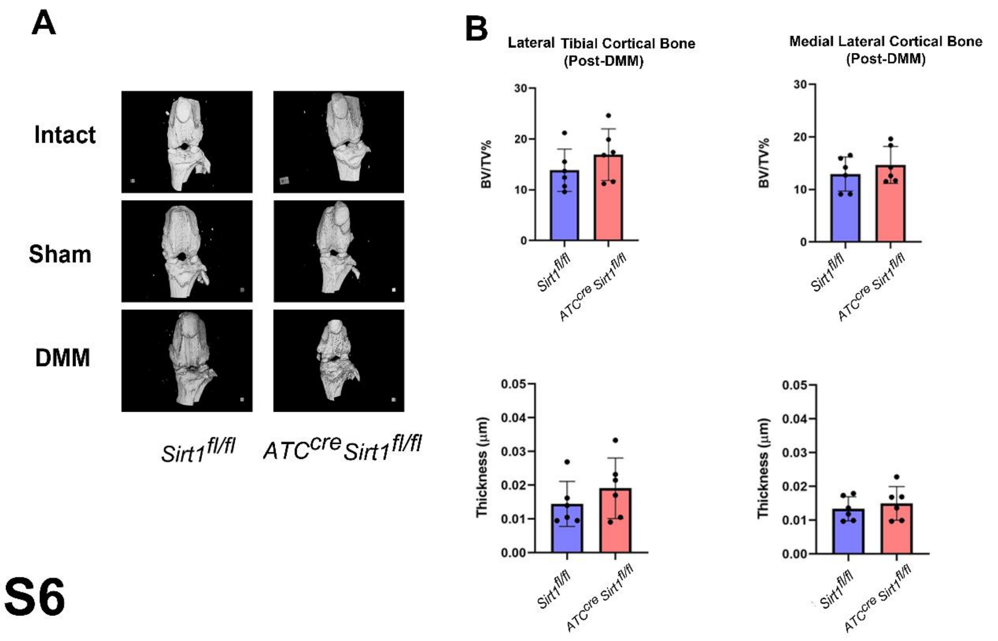

<u>S6:</u> Micro-CT joint reconstruction and analysis post-DMM. (**A)** 3D µCT joint reconstructions of Intact non-operated mice, SHAM-operated mice, DMM-operated mice. 3D images demonstrate abnormal bone morphology post-DMM when compared to SHAM and Intact. (**B**) Tibial subchondral bone plate was selected as area of interest to perform analysis in for *Sirt1*$^{fl/fl}$ and *ATC*^{cre}*Sirt1*$^{fl/fl}$. Upper two graphs show bone volume over tissue volume, BV/TV (%) post-DMM for *Sirt1*$^{fl/fl}$ and *ATC*^{cre}*Sirt1*$^{fl/fl}$. Lower two graphs represent subchondral bone plate thickness (µm) post DMM for *Sirt1*$^{fl/fl}$ and *ATC*^{cre}*Sirt1*$^{fl/fl}$. Results showed no significant differences in subchondral bone

Batshon G, *et al. Ann Rheum Dis* 2020; 79:1370–1380. doi: 10.1136/annrheumdis-2020-217072

***Supplemental Information**: Batshon et al., 2020*

plate volume and thickness between genotype groups post-DMM. (n=6, for each group).

Figure S7

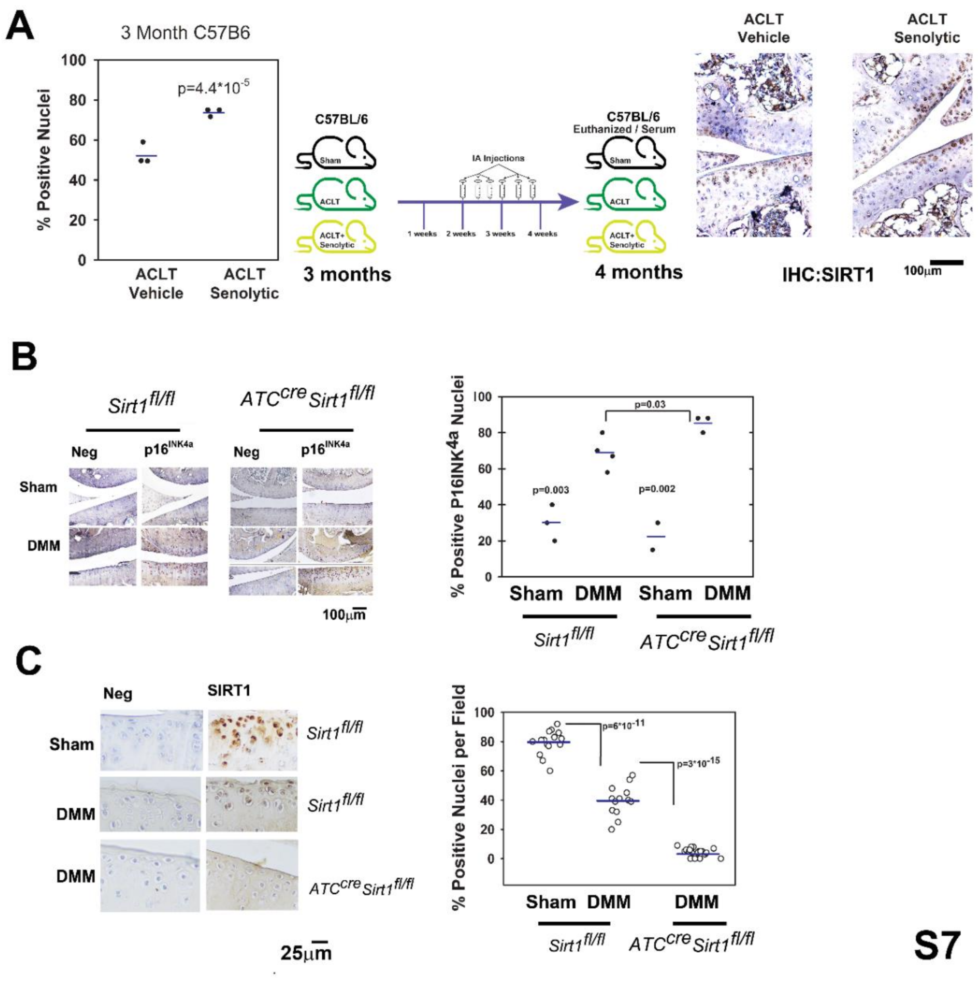

<u>S7</u>: Sirt1 ablation contributes to cartilage senescence in post-traumatic OA. C57B6 Mice at age 3 months were subjected to ACLT procedure and treated with saline or UBX0101 intra-articularly (similar to protocol by Jeon *et*

Batshon G, *et al. Ann Rheum Dis* 2020; 79:1370–1380. doi: 10.1136/annrheumdis-2020-217072

***Supplemental Information**: Batshon et al., 2020*

al., [4]). (**A**) Immunohistochemistry for N-terminal SIRT1 of ACLT-Senolytic or ACLT-Vehicle treated mice (n=3, right image). Left panel shows quantification of SIRT1 positive nuclei from total cell population per field, in immunohistochemically sections. (**B**) Immunohistochemistry for $p16^{INK4a}$ shows it is increased following DMM in $Sirt1^{fl/fl}$ ($Sirt1^{fl/fl}$, sham n=3, DMM n=4) and $ATC^{cre}Sirt1^{fl/fl}$ ($ATC^{cre}Sirt1^{fl/fl}$, sham n=2, DMM n=3). Based on quantification of positive nuclei, absence of Sirt1 in cartage further enhanced $p16^{INK4a}$ positive nuclei. (**C**) Immunohistochemistry for CT-SIRT1 in $Sirt1^{fl/fl}$ ($Sirt1^{fl/fl}$, sham n=3, DMM n=3) show reduced Sirt1 with DMM, which is similarly absent in $ATC^{cre}Sirt1^{fl/fl}$ transgene post-DMM ($ATC^{cre}Sirt1^{fl/fl}$, DMM n=4).

Batshon G, *et al. Ann Rheum Dis* 2020; 79:1370–1380. doi: 10.1136/annrheumdis-2020-217072

***Supplemental Information**: Batshon et al., 2020*

Figure S8

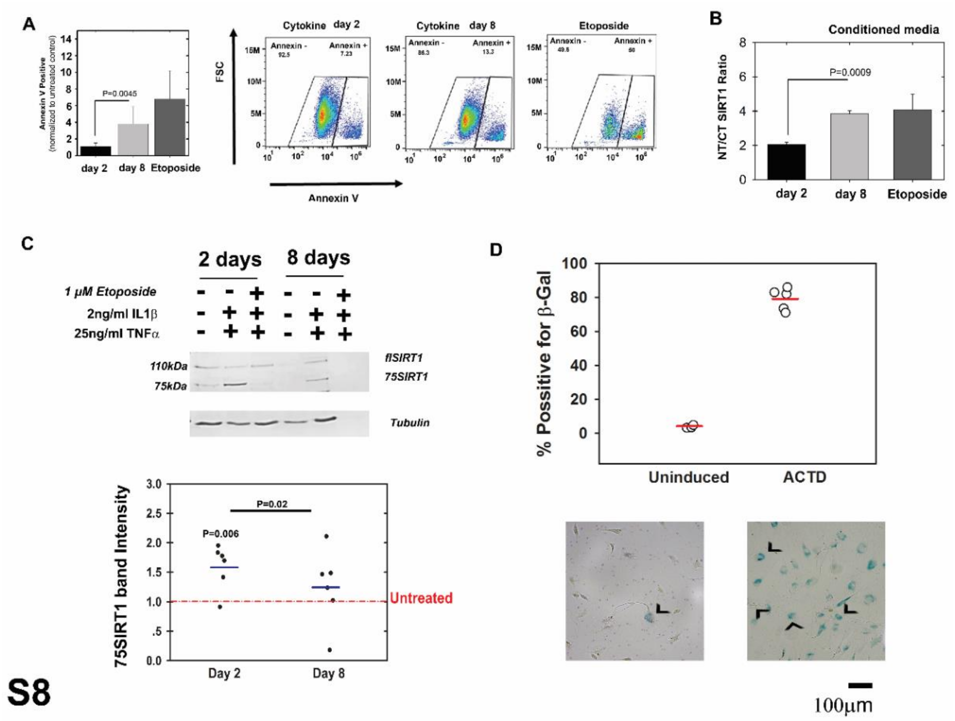

<u>S8</u>: NT/CT SIRT1 fragments in conditioned media are generated from cytokine-stimulated non-senescent chondrocytes. (**A**) Human chondrocytes were treated for 2 or 8 days with cytokines (25 ng/ml TNFα and 2 ng/ml IL1β, denoted as "cytokine"). Analysis of apoptotic cell levels using annexin V staining and Fluorescence Activated Cell Sorting (FACS), (n=5). Representative FACS diagrams are presented beneath the graph (n=5), (**B**) Indirect ELISA of conditioned media from chondrocytes treated as above for 2 and 8 days before harvested and analyzed via ELISA assay. Etoposide was used as a positive control. (**C**) Immunoblot of cell-derived protein extracts from A for NT-SIRT1, showing the full-length and 75SIRT1 variant, (n=5). (**D**). Quantification of blue-stained cells following β-galactosidase staining procedure, as detailed in materials and methods. Bar indicates average. Statistical significance was determined for $p<0.05$ via Mann and Whitney test.

Batshon G, *et al. Ann Rheum Dis* 2020; 79:1370–1380. doi: 10.1136/annrheumdis-2020-217072
